# Navy Bean and Rice Bran Intake Alters the Plasma Metabolome of Children at Risk for Cardiovascular Disease

**DOI:** 10.3389/fnut.2017.00071

**Published:** 2018-01-19

**Authors:** Katherine J. Li, Erica C. Borresen, NaNet Jenkins-Puccetti, Gary Luckasen, Elizabeth P. Ryan

**Affiliations:** ^1^Nutrition and Toxicology Laboratory, Department of Environmental and Radiological Health Sciences, College of Veterinary Medicine and Biomedical Sciences, Colorado State University, Fort Collins, CO, United States; ^2^Medical Center of the Rockies, University of Colorado Health Research – Northern Region, Loveland, CO, United States

**Keywords:** navy bean, rice bran, metabolomics, children, cholesterol, cardiovascular disease

## Abstract

Abnormal cholesterol in childhood predicts cardiovascular disease (CVD) risk in adulthood. Navy beans and rice bran have demonstrated efficacy in regulating blood lipids in adults and children; however, their effects on modulating the child plasma metabolome has not been investigated and warrants investigation. A pilot, randomized-controlled, clinical trial was conducted in 38 children (10 ± 0.8 years old) with abnormal cholesterol. Participants consumed a snack for 4 weeks containing either: no navy bean or rice bran (control); 17.5 g/day cooked navy bean powder; 15 g/day heat-stabilized rice bran; or 9 g/day navy beans and 8 g/day rice bran. Plasma metabolites were extracted using 80% methanol for global, non-targeted metabolic profiling *via* ultra-high performance liquid-chromatography tandem mass spectrometry. Differences in plasma metabolite levels after 4 weeks of dietary intervention compared to control and baseline were analyzed using analysis of variance and Welch’s *t*-tests (*p* ≤ 0.05). Navy bean and/or rice bran consumption influenced 71 plasma compounds compared to control (*p* ≤ 0.05), with lipids representing 46% of the total plasma metabolome. Significant changes were determined for 18 plasma lipids in the navy bean group and 10 plasma lipids for the rice bran group compared to control, and 48 lipids in the navy bean group and 40 in the rice bran group compared to baseline. These results support the hypothesis that consumption of these foods impact blood lipid metabolism with implications for reducing CVD risk in children. Complementary and distinct lipid pathways were affected by the diet groups, including acylcarnitines and lysolipids (navy bean), sphingolipids (rice bran), and phospholipids (navy bean + rice bran). Navy bean consumption decreased free fatty acids associated with metabolic diseases (palmitate and arachidonate) and increased the relative abundance of endogenous anti-inflammatory lipids (endocannabinoids, N-linoleoylglycine, 12,13-diHOME). Several diet-derived amino acids, phytochemicals, and cofactors/vitamins with cardioprotective properties were increased compared to control and/or baseline, including 6-oxopiperidine-2-carboxylate (1.87-fold), *N*-methylpipecolate (1.89-fold), trigonelline (4.44- to 7.75-fold), *S*-methylcysteine (2.12-fold) (navy bean), salicylate (2.74-fold), and pyridoxal (3.35- to 3.96-fold) (rice bran). Findings from this pilot study support the need for investigating the effects of these foods for longer durations to reduce CVD risk. Trial registration: clinicaltrials.gov (identifier NCT01911390).

## Introduction

Cardiovascular disease (CVD) is the leading cause of death in the United States and globally (1). Childhood and adolescence marks a critical period for the emergence of CVD risk factors, and several epidemiological studies have indicated that abnormal cholesterol levels early in life predicts atherosclerosis and CVD later in life (2–7). Data from the National Health and Nutrition Examination Survey 2011–2014 reveal that approximately 21% of children and adolescents in the US have at least one abnormal serum cholesterol measure (8, 9). In addition, histopathological evidence for atherosclerosis has also been found in the arterial walls of children as young as 3 years old (10). A compelling rationale exists to sustainably control blood lipids during childhood to prevent or delay the development of life-threatening cardiovascular events later in life.

Lifestyle modifications in diet and physical activity can protect against CVD across the lifespan, and plant-based diets high in stanols/sterols and soluble fiber have been recognized by the National Cholesterol Education Program as an important strategy to reduce or control cholesterol (9, 11). Proper nutrition in childhood can lend long-term protection against multiple risk factors for CVD not only *via* controlling blood lipids, but also by regulating the underlying cellular processes that drive metabolic diseases. Metabolomics is an emerging tool for dietary intervention trials and pediatric research that provides insight into the complex relationships between dietary exposures, host digestion and gut microbial metabolism (12–16). Through non-targeted metabolomics approaches, all metabolites (in theory) within a biological sample can be identified in a non-biased manner, to reveal novel insights and biomarkers (17).

Cooked dry beans (a legume) and rice bran (a whole grain component) are rich sources of proteins, lipids, vitamins, fiber, and phytochemicals that have favorable impacts on intestinal and cardiovascular health (18–22). Increased consumption of navy beans and rice bran have demonstrated efficacy in lowering serum cholesterol levels in experimental animals (23, 24), hypercholesterolemic adults (19, 25, 26), as well as modulating high-density lipoprotein (HDL) and low-density lipoprotein (LDL) cholesterol in some children after 4 weeks of consumption (27). However, the effects of navy bean and rice bran consumption on the plasma metabolome of children with abnormal cholesterol have not been previously evaluated. A global, non-targeted metabolomics analysis was applied to reveal the network of metabolic pathways altered by 4 weeks of daily intake with navy bean, rice bran, or a combination of navy bean + rice bran when compared to a control at 4 weeks, or compared to their respective baseline. We hypothesized that navy bean and rice bran consumption favorably modulates multiple metabolic pathways associated with cholesterol-lowering in children. Identification of bioactive food compounds with cardioprotective properties will elucidate important mechanisms by which navy beans and rice bran exert their health-promoting effects and may uncover candidate dietary biomarkers of intake.

## Materials and Methods

### Study Participants

Healthy children aged 8–13 years old were recruited from a school-based program (Healthy Hearts) in Northern Colorado, as previously described (27, 28). These children underwent cholesterol screening and were included on the basis of abnormal cholesterol levels that put them at high risk for future CVD. Criteria for abnormal cholesterol included either: total cholesterol ≥ 180 mg/dL and HDL < 60 mg/dL; LDL ≥ 100 mg/dL and HDL < 60 mg/dL; or non-HDL > 100 mg/dL; and HDL < 60 mg/dL. Children with ongoing medical illnesses, taking medications, or with food allergies or major dietary restrictions, were excluded from participation. This study was carried out in accordance with the recommendations of local and national guidelines, and study protocols were approved by the University of Colorado Health-North Institutional Review Board (Protocol 13-1263) and the Colorado State University Research Integrity and Compliance Review Office (Protocol 13-4390). Prior to the start of the trial, written informed consent were obtained from guardians and written informed assent from all participants in accordance with the Declaration of Helsinki. This trial was registered at clinicaltrials.gov under NCT01911390.

### Dietary Interventions

**Figure 1:**
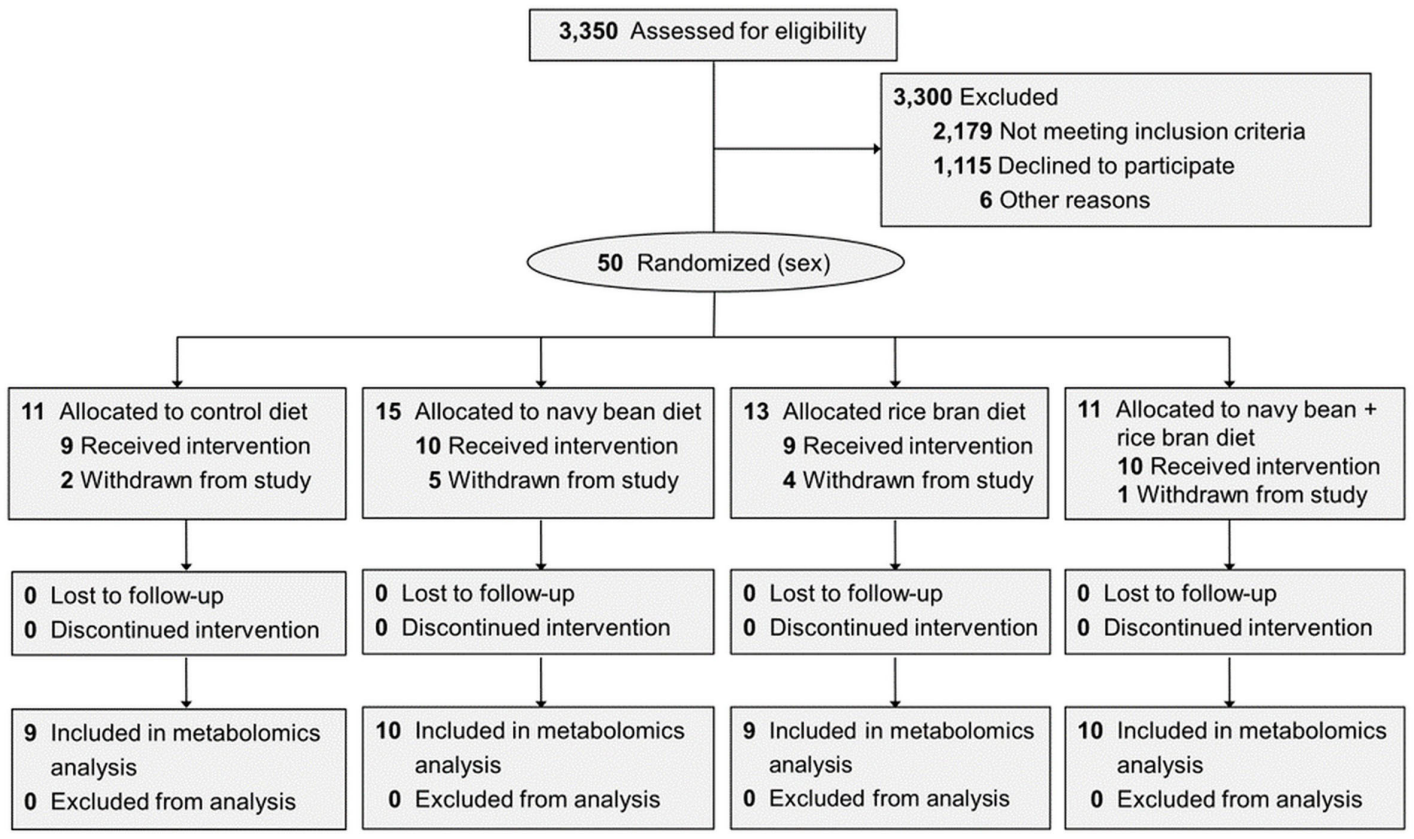
Participants in each dietary intervention group and the plasma samples used for metabolome analysis. CONSORT diagram shown in Borresen et al. (27). Reasons for withdrawal included: noncompliance to the study protocol (*n* = 5), declined to participate (*n* = 6), and gastrointestinal issues (*n* = 1).

Eligible participants were computer-randomized by sex into one of four dietary intervention groups. All participants received a muffin or smoothie daily for 4 weeks that had (1) no navy beans or rice bran (control, 0 g/day), (2) 17.5 g/day cooked navy beans, (3) 15 g/day heat-stabilized rice bran, or (4) a combination of 9 g/day navy bean and 8 g/day rice bran (Figure 1). The amounts of navy bean and/or rice bran in the navy bean, rice bran, and navy bean + rice bran groups provided 55, 48, and 53 kcal/day, respectively (27). Study-provided foods were coded to ensure participant blinding. Cooked navy bean powder (VegeFull™) was provided by Archer Daniels Midland Edible Bean Specialties, Inc. (Decatur, IL, USA), and heat-stabilized rice bran was provided by the US Department of Agriculture-Agricultural Research Service Dale Bumpers National Rice Research Center (Stuttgart, AR, USA). All study foods were prepared in a commercial kitchen with quality control measures at Colorado State University as previously described (27).

### Blood Sample Collection

Fasting blood samples were collected from the participants by venipuncture at baseline and week 4. Blood samples were collected into 4-mL sodium citrate cell preparation tubes (BD Biosciences, Franklin Lakes, NJ, USA) and centrifuged at 1,500 relative centrifugal force at room temperature for 30 min to separate red and white cells from plasma. Plasma was aliquoted (0.5 mL) and frozen at −80°C until metabolomics analysis.

### Plasma Metabolite Extraction, Identification, and Metabolome Analysis

Metabolon, Inc. (Durham, NC, USA) performed plasma metabolite extraction and non-targeted global metabolic profiling on all samples *via* ultra-high performance liquid-chromatography tandem mass spectrometry (UPLC–MS/MS). Data were accessioned into Metabolon’s Library Information Management System and assigned a unique identifier associated with the participant study codes that tracked all clinical trial information.

Plasma was prepared using the automated MicroLab STAR^®^ system (Hamilton Company). To remove protein and recover small molecules, the sample was precipitated with methanol and vigorously shaken for 2 min (Glen Mills GenoGrinder 2000) followed by centrifugation. The resulting extract was analyzed by reverse phase UPLC-MS/MS with positive and negative ion mode electrospray ionization and hydrophilic interaction liquid chromatography (HILIC) with negative ion mode electrospray ionization. Extracts were placed briefly on a TurboVap^®^ (Zymark) automated evaporation system to remove remaining organic solvent and stored in liquid nitrogen prior to analysis.

UPLC–MS/MS was completed using a Waters ACQUITY UPLC and a Thermo Scientific Q-Exactive high resolution/accurate mass spectrometer interfaced with a heated electrospray ionization source and Orbitrap mass analyzer operated at 35,000 mass resolution. The dried sample was reconstituted in solvents containing a series of standards at fixed concentrations. Extracts were analyzed using acidic positive ion conditions optimized for hydrophilic or hydrophobic compounds by gradient eluting from a dedicated C18 column (Waters UPLC BEH C18-2.1 mm × 100 mm, 1.7 µm) using water, methanol, 0.05% perfluoropentanoic acid, and 0.1% formic acid (hydrophilic), or methanol, acetonitrile, water, 0.05% perfluoropentanoic acid, and 0.01% formic acid (hydrophobic). A basic negative ion extract was gradient eluted from a separate C18 column using methanol, water, and 6.5 mM ammonium bicarbonate at pH 8, and the remaining extract was analyzed *via* negative ionization following gradient elution from a HILIC column (Waters UPLC BEH Amide 2.1 mm × 150 mm, 1.7 µm) using water, acetonitrile, and 10 mM ammonium formate at pH 10.8. The MS analysis alternated between MS and data-dependent MS^n^ scans using dynamic exclusion (70–1,000 *m/z*).

Raw data were extracted and peak-identified by Metabolon. Identity of the compounds were confirmed by comparison to an internal library of over 3,300 entries of purified standards or recurrent unknown entities maintained by Metabolon, based on the retention time/index, *m/z*, and chromatographic data. Metabolites were quantified in terms of relative abundance and a scaled relative abundance was calculated for each metabolite by dividing its raw abundance by the median value of the metabolite across the dataset. Metabolite ratios were calculated by dividing the median-scaled abundance of each metabolite in the navy bean, rice bran, and navy bean + rice bran groups at 4 weeks by that of the control group at 4 weeks, or by their respective baseline at week 0, to identify metabolites that were significantly different between groups (fold differences) or within groups (fold changes).

### Food Metabolite Extraction and Identification

Non-targeted metabolomics on the navy bean powder and heat-stabilized rice bran (each 100 mg) was performed by Metabolon. Food metabolites were extracted with 80% methanol and analyzed by UPLC-MS/MS and gas-chromatography mass spectrometry (GC-MS) in the positive and negative ionization mode platforms. UPLC-MS/MS was performed using the same methods as described above. For GC-MS, the food extracts were derivatized under nitrogen using bistrimethyl-silyltrifluoroacetamide and separated on a 5% diphenyl/95% dimethyl polysiloxane-fused silica column (20 m × 0.18 mm ID; 0.18 µm film thickness) using helium as carrier gas and a temperature ramp from 60 to 340°C in a 17.5-min period. Internal standards (250 ng each of amylbenzene, 1-phenylhexane, 1-phenyloctane, 1-phenyldecane, 1-phenyldodecane, hexadecylbenzene, octadecylbenzene, tetradecylbenzene and 2,6-di-tert-butyl-4-methylphenol) were added to each sample. Samples were analyzed on a Thermo-Finnigan Trace DSQ fast-scanning single-quadrupole mass spectrometer using electro impact ionization (EI) and operated at unit mass resolving power (scan range 50–750 *m/z*). Raw data were peak-identified as described above and raw counts of each metabolite were quantified using area-under-the-curve.

To ascertain whether metabolites identified in plasma could be of dietary origin (exclusively or in part), the full list of plasma metabolites identified in each dietary group after 4 weeks of consumption was cross-referenced with metabolites identified in the food metabolome.

### Metabolic Pathway Visualizations

Metabolic pathway visualizations were generated using the Metabolync™ plug-in for Cytoscape (Version 2.8.3). Pathway enrichment scores for metabolic subpathways were calculated using the following equation, where *k* represents the number of significant metabolites in a pathway, *m* represents the total number of identified metabolites in that pathway, *n* represents the total number of significant metabolites in the complete dataset, and *N* represents the total number of identified metabolites in the complete dataset:

$$\frac{k/m}{n/N}.$$

Pathways with enrichment scores greater than one indicated that the metabolic pathway contained a higher number of metabolites with statistically significant fold-differences or fold-changes compared to other pathways in the study.

### Statistical Analysis

Power calculation for this pilot study was based on effect sizes reported in a previous rice bran dietary intervention metabolomics study (29, 30). Two-way analysis of variance (ANOVA) and Welch’s two-sample *t*-tests were used to determine statistical significance between groups, and ANOVA with repeated measures was used to determine statistical significance within groups (*p* ≤ 0.05). Metabolites that showed statistical difference at baseline between navy bean, rice bran, or navy bean + rice bran groups and control were removed from the between-group analyses. An estimate of the false discovery rate was calculated (*q*-value) to take into account the multiple comparisons relevant to metabolomics investigations.

## Results

### Effects of Navy Bean and/or Rice Bran on Modulating the Nutritional Metabolome of Children at Risk for CVD

**Figure 2:**
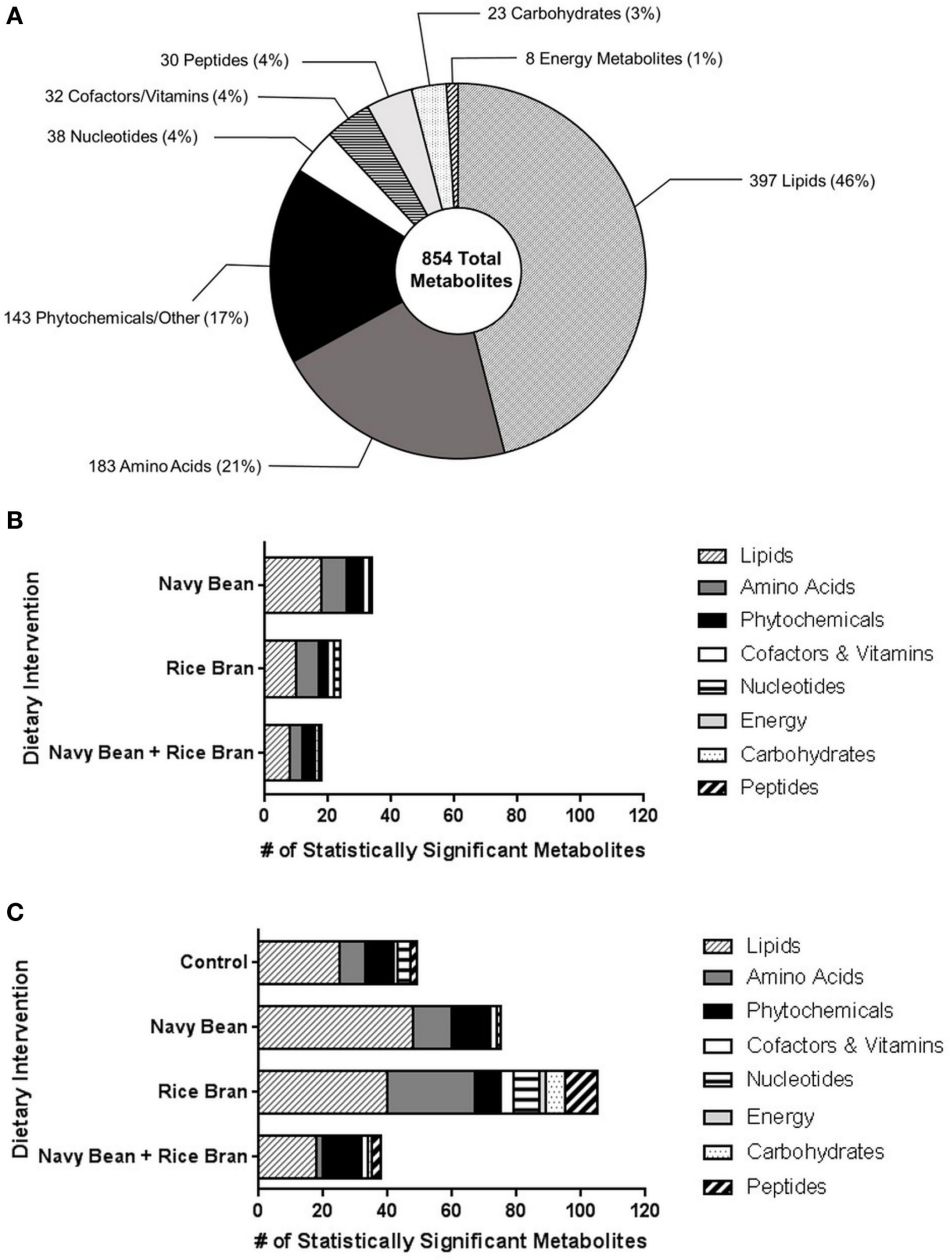
Nutritional metabolome of children modulated by navy bean and/or rice bran consumption. **(A)** Total metabolite profile identified from plasma of children across all dietary groups. Total numbers of significantly modulated metabolites (increased and decreased, *p* ≤ 0.05) across eight metabolite classes after 4 weeks of navy bean, rice bran, or navy bean + rice bran consumption **(B)** compared to control group at 4 weeks or **(C)** compared to respective baseline.

Thirty-eight children (10 ± 0.8 years old) enrolled and randomized to a dietary intervention successfully completed the trial and were analyzed for changes in the plasma metabolome (Figure 1). Across the four dietary interventions, a total of 854 metabolites from eight chemical classes were identified in plasma, of which 397 were classified as lipids, 183 amino acids, 143 phytochemicals, 38 nucleotides, 32 cofactors/vitamins, 30 peptides, 23 carbohydrates, and 8 energy pathway metabolites (Figure 2A). Navy bean and/or rice bran consumption resulted in statistically significant modulation of metabolites from all eight metabolic pathway classifications (*p* ≤ 0.05) (Figures 2B,C). Compared to control at week 4, the total number of significantly altered metabolites in each diet group were 34 (navy bean), 24 (rice bran), and 18 (navy bean + rice bran) (Figure 2B). Compared to their respective baseline, the total number of significantly altered plasma metabolites were 49 (control), 68 (navy bean), 74 (rice bran), and 37 (navy bean + rice bran) (Figure 2C). Lipid, amino acid, and phytochemical pathways contained the largest total number of significantly modulated metabolites, with comparatively fewer significant metabolites identified from the cofactor/vitamin, nucleotide, energy, carbohydrate, and peptide classes.

### Differences in Plasma Lipid Metabolites following Navy Bean and/or Rice Bran Consumption

**Figure 3:**
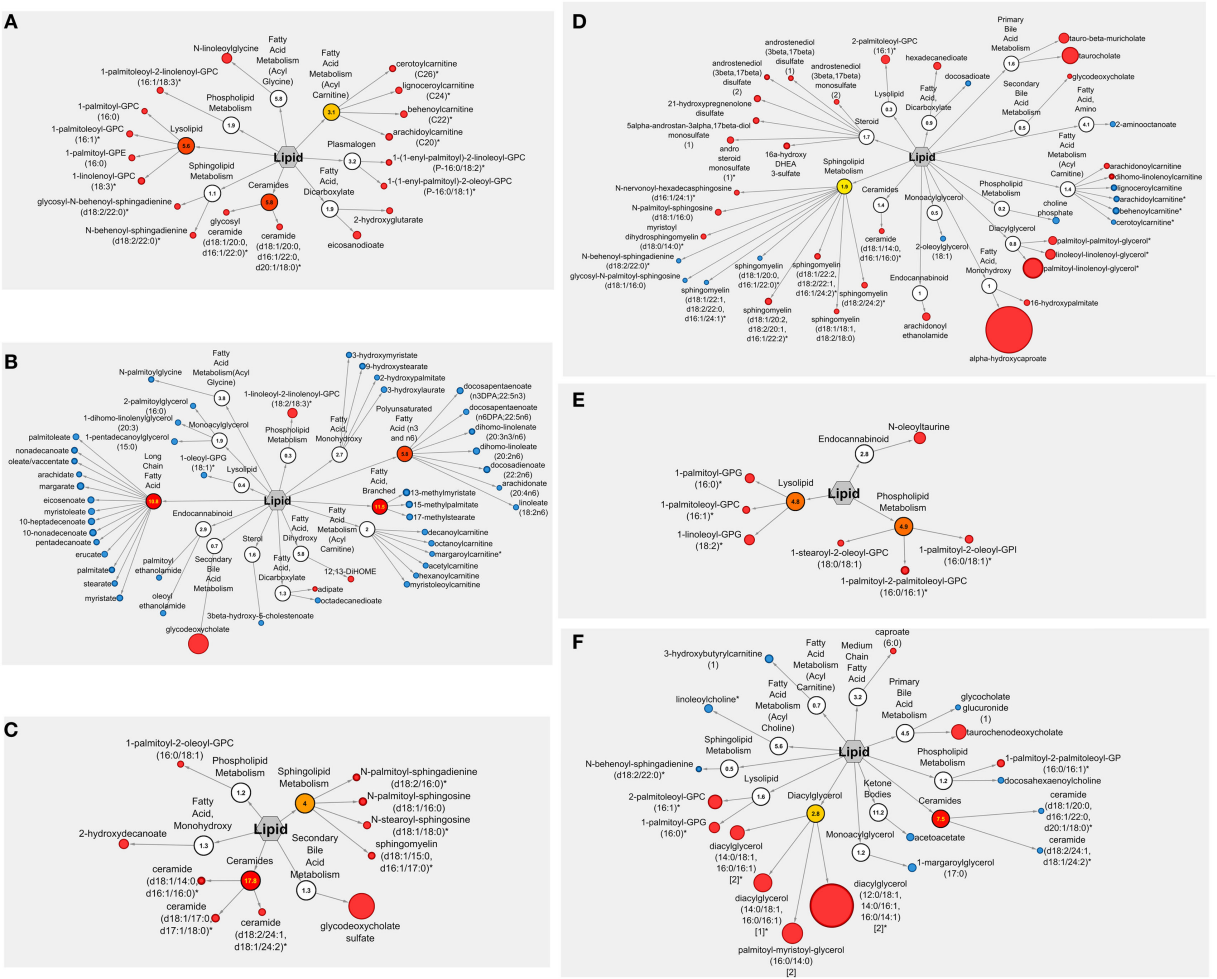
Cytoscape pathway visualizations of lipid metabolites modulated by navy bean, rice bran, or navy bean + rice bran consumption. Lipid metabolites modulated by navy bean, rice bran, or navy bean + rice bran consumption for 4 weeks compared to control is shown in panels **A,C,E** respectively. Lipid metabolites modulated by navy bean, rice bran, or navy bean + rice bran consumption for 4 weeks compared to respective baseline is shown in panels **B,D,F** respectively. Nodes in red and blue represent significantly increased and decreased metabolites, respectively, compared to control or baseline (*p* ≤ 0.05). For each metabolite, the node diameter is proportional to the magnitude of the fold difference/change. Numerical values within nodes indicate the calculated pathway enrichment score. Pathways with enrichment scores greater than 1 were defined as being important contributors to overall dietary group differences (visualized in red/yellow).

Consumption of navy bean and/or rice bran resulted in a global increase in lipid metabolites compared to control at 4 weeks (Table 1). Submetabolic pathways of lipids modulated by navy bean and/or rice bran consumption were distinct and complementary, as shown in Table 1 and visualized in Figures 3A,C,E. Navy bean consumption significantly increased 18 lipids compared to control at 4 weeks, including 4 acylcarnitine (1.37- to 1.47-fold difference), 4 lysolipid (1.21- to 1.45-fold difference), and 2 ceramide (1.24- to 1.28-fold difference) pathway metabolites (Table 1). Significant increases in several plasmalogens, acylglycines, phospholipids, dicarboxylate fatty acids, and sphingolipids were also determined. Rice bran consumption significantly increased 10 lipids compared to control at 4 weeks, including 4 sphingolipid metabolites (1.28- to 1.42-fold difference), 3 ceramide metabolites (1.28- to 1.42-fold difference), the secondary bile acid glychodeoxycholate sulfate (4.37-fold difference), as well as several phospholipid and monohydroxy fatty acids (Table 1). Consumption of navy bean + rice bran significantly increased seven lipids compared to control at 4 weeks, which included three lysolipid metabolites (1.39- to 1.90-fold difference), three phospholipid metabolites (1.21- to 1.54-fold difference), and the endocannabinoid *N*-oleoyltaurine (2.15-fold difference) (Table 1). One lipid metabolite (carnitine) was decreased following navy bean + rice bran consumption compared to control at 4 weeks. Overall, fewer lipid metabolites were affected by the navy bean + rice bran intervention (7) than for navy bean (10) or rice bran (18) separately.

**Table 1:** Modulated plasma lipid metabolites following navy bean and/or rice bran consumption for 4 weeks compared to control.^a^

Metabolic subpathway	Metabolite	Fold Difference vs. Control^b^		
		Navy bean	Rice bran	Navy bean + rice bran
Fatty acid, dicarboxylate	2-hydroxyglutarate	1.32↑*	1.02	1.12
	Eicosanodioate	1.63↑*	1.09	1.27

Fatty acid metabolism (acyl glycine)	*N*-linoleoylglycine	2.05↑*	1.39	1.72

Fatty acid metabolism (acyl carnitine)	Arachidoylcarnitine	1.47↑*	1.17	1.24
	Behenoylcarnitine	1.44↑*	1.12	1.22
	Lignoceroylcarnitine	1.40↑*	1.19	1.21
	Cerotoylcarnitine	1.37↑**	1.22	1.27

Carnitine metabolism	Carnitine	0.93	0.92	0.87↓*

Fatty acid, monohydroxy	2-Hydroxydecanoate	1.22	1.70↑*	1.26

Endocannabinoid	*N*-oleoyltaurine	1.60	1.36	2.15↑*

Phospholipid metabolism	1-Palmitoyl-2-oleoyl-GPC	1.17	1.16↑*	1.24
	1-Stearoyl-2-oleoyl-GPC	1.18	1.13	1.21↑*
	1-Palmitoyl-2-palmitoleoyl-GPC	1.16	1.15	1.54↑**
	1-Palmitoyl-2-oleoyl-GPI	1.06	1.21	1.51↑*
	1-Palmitoleoyl-2-linolenoyl-GPC	1.35↑*	1.14	1.52

Lysolipid	1-Palmitoyl-GPC	1.21↑*	1.14	1.19
	1-Palmitoleoyl-GPC	1.35↑*	1.21	1.39↑*
	1-Linolenoyl-GPC	1.45↑**	1.04	1.42
	1-Palmitoyl-GPE	1.33↑*	1.07	1.26
	1-Palmitoyl-GPG	1.09	1.09	1.75↑*
	1-Linoleoyl-GPG	1.26	1.33	1.90↑*

Plasmalogen	1-(1-Enyl-palmitoyl)-2-oleoyl-GPC	1.28↑*	1.13	1.15
	1-(1-Enyl-palmitoyl)-2-linoleoyl-GPC	1.27↑**	1.10	1.12

Sphingolipid metabolism	*N*-palmitoyl-sphingadienine (d18:2/16:0)	1.15	1.30↑**	1.19
	*N*-behenoyl-sphingadienine (d18:2/22:0)	1.28↑*	1.37	1.15
	Sphingomyelin (d18:1/15:0, 16:1/17:0)	1.13	1.28↑**	1.10
	*N*-palmitoyl-sphingosine (d18:1/16:0)	1.23	1.42↑**	1.37
	*N*-stearoyl-sphingosine (d18:1/18:0)	1.13	1.33↑*	1.12
	glycosyl-N-behenoyl-sphingadienine (d18:2/22:0)	1.25↑*	1.18	1.19

Secondary bile acid metabolism	Glycodeoxycholate sulfate	5.70	4.37↑*	4.12

Ceramides	Ceramide (d18:1/14:0, d16:1/16:0)	1.12	1.37↑**	1.23
	Ceramide (d18:1/17:0, d17:1/18:0)	1.11	1.42↑**	1.10
	Ceramide (d18:1/20:0, d16:1/22:0, d20:1/18:0)	1.28↑*	1.32	1.17
	Ceramide (d18:2/24:1, d18:1/24:2)	1.12	1.28↑*	1.17
	Glycosyl ceramide (d18:1/20:0, d16:1/22:0)	1.24↑*	1.30	1.32

*GPC, glycerophosphocholine; GPE, glycerophosphoethanolamine; GPG, glycerophosphoglycerol; GPI, glycerophosphoinositol*.

*^a^Metabolites that were detected in plasma but non-significant (*p* > 0.05) in dietary groups compared to respective baseline are not presented in this table*.

^b^Values presented are mean metabolite ratios/fold differences. Statistically significantly increased (↑) fold differences are bolded and highlighted in red, and statistically significantly decreased (↓) fold differences are bolded and highlighted in blue (*p ≤ 0.05; **p ≤ 0.01; ***p ≤ 0.001).

Numerous lipid metabolites were also significantly modulated by each of the dietary groups at 4 weeks compared to their respective baseline, with the largest effect observed in the navy bean group (48 significantly modulated lipid metabolites) (Table 2). Similar to the between-group analyses, submetabolic pathways of lipids affected by navy bean and/or rice bran consumption compared to their baseline levels were distinct and complementary (Figures 3B,D,F). Table 2 reveals that navy bean consumption decreased 44 lipids and increased 4 lipids compared to baseline. Lipids with decreased relative abundance included 14 long-chain fatty acids (0.63- to 0.80-fold change), 8 polyunsaturated fatty acids (0.66- to 0.86-fold change), 6 acyl carnitines (0.75- to 0.84-fold change), 4 monohydroxy fatty acids (0.72- to 0.82-fold change), 3 branched fatty acids (0.63- to 0.70-fold change), along with several endocannabinoid, monoacylglycerol, lysolipid, sterol, dicarboxylate fatty acid, and acyl glycine metabolites. Lipids with increased relative abundance included the dicarboxylate fatty acid adipate (1.12-fold change), the dihydroxy fatty acid 12,13-DiHOME (1.36-fold change), the phospholipid 1-linoleoyl-2-linolenoyl-GPC (18:2/18:3) (2.11-fold change), and the secondary bile acid metabolite glycodeoxycholate (4.27-fold change). Following rice bran consumption, significant decreases in 12 and increases in 28 lipid metabolites were determined compared to baseline (Table 2). Classes of modulated lipid metabolites included decreases in 4 acyl carnitines (0.65- to 0.89-fold change) and 4 sphingolipids (all 0.91-fold change), and increases in 7 sphingolipids (1.19- to 1.55-fold change), 6 steroids (1.20- to 1.52-fold change), 4 diacylglycerols (1.88- to 4.44-fold change), 3 primary bile acids (taurocholate; 1.54-fold change, taurochenodeoxycholate; 3.65-fold change, and tauro-beta-muricholate; 2.29-fold change) and a secondary bile acid (glycodeoxycholate; 1.10-fold change). For the navy bean + rice bran group, 9 lipid metabolites were decreased compared to baseline, including 2 ceramides (0.87- to 0.90-fold change), the primary bile acid glycocholate glucuronide (0.93-fold change change), and singular decreases in acyl carnitine, ketone body, phosholipid, monoacylglycerol, sphingolipid, and acyl choline metabolites. Of the suite of nine metabolites increased following consumption of navy bean + rice bran compared to baseline, the most notable includes an increase in four diacylglyerols (2.70- to 8.03-fold change) and the primary bile acid taurochenodeoxycholate (2.81-fold change). Several increases in medium chain fatty acid, phospholipid, and lysolipid metabolites were also determined (Table 2). Interestingly, comparison of the control group at 4 weeks to its respective baseline also revealed 22 decreased and 3 increased lipid metabolites, including 6 polyunsaturated fatty acids (0.64- to 0.79-fold change), 5 monoacylglycerols (0.71- to 0.85-fold change), 6 steroids (0.73- to 0.85-fold change), and modulations in several acylcarnitine, carnitine, endocannabinoid, plasmalogen, and secondary bile acid metabolites (Table 2).

**Table 2:** Modulated plasma lipid metabolites following navy bean and/or rice bran consumption for 4 weeks compared to baseline^a^.

Metabolic subpathway	Metabolite	Fold change vs. baseline^b^			
		Control	Navy bean	Rice bran	Navy bean + rice bran
Medium chain fatty acid	Caproate	1.05	1.00	0.96	1.17↑*

Long-chain fatty acid	Myristate	0.91	0.70↓*	1.47	1.09
	Myristoleate	1.03	0.69↓*	1.56	1.39
	Pentadecanoate	0.85	0.76↓**	1.18	0.94
	Palmitate	0.91	0.74↓**	1.19	0.90
	Palmitoleate	0.90	0.72↓*	1.64	1.27
	Margarate	0.78	0.63↓**	1.25	0.89
	10-Heptadecenoate	0.85	0.66↓*	1.47	1.02
	Stearate	0.89	0.76↓**	1.08	0.91
	Nonadecanoate	0.88	0.71↓**	1.14	0.95
	10-Nonadecenoate	0.79	0.64↓**	1.20	0.89
	Arachidate	0.86	0.80↓**	0.99	0.98
	Eicosenoate	0.84	0.68↓**	1.13	0.88
	Erucate	0.77	0.73↓*	0.91	1.23
	Oleate/vaccenate	0.87	0.74↓**	1.12	0.95

Polyunsaturated fatty acid (n3 and n6)	Docosapentaenoate (n3; 22:5n3)	0.64↓**	0.68↓**	1.30	0.94
	Docosahexaenoate (22:6n3)	0.74↓*	0.83	1.18	1.02
	Linoleate (18:2n6)	0.86	0.80↓*	1.20	0.92
	Dihomo-linolenate (20:3n3 or n6)	0.76↓*	0.74↓**	1.20	0.97
	Arachidonate (20:4n6)	0.78↓**	0.86↓*	1.13	0.97
	Adrenate (22:4n6)	0.64↓*	0.68↓*	1.86	1.23
	Docosapentaenoate (n6; 22:5n6)	0.79↓*	0.77↓*	1.20	1.04
	Docosadienoate (22:2n6)	0.84	0.67↓**	1.17	0.89
	Dihomo-linoleate (20:2n6)	0.73	0.66↓*	1.24	0.90

Fatty acid, branched	13-Methylmyristate	0.90	0.63↓**	1.33	0.99
	15-Methylpalmitate	0.93	0.66↓**	1.31	0.97
	17-Methylstearate	0.97	0.70↓**	1.18	0.96

Fatty acid, dicarboxylate	Adipate	1.06	1.12↑**	1.02	1.02
	Hexadecanedioate	0.87	0.91	1.70↑*	0.93
	Octadecanedioate	0.87	0.80↓*	1.44	0.83
	Docosadioate	1.08	1.44	0.79↓*	1.29

Fatty acid, amino	2-Aminooctanoate	1.06	1.22	0.78↓*	1.00

Fatty acid metabolism (acyl glycine)	*N*-palmitoylglycine	0.80	0.76↓*	1.09	1.17

Fatty acid metabolism (acyl carnitine)	Acetylcarnitine	1.08	0.80↓*	1.13	0.85
	3-Hydroxybutyrylcarnitine	2.38	0.86	1.27	0.63↓*
	Hexanoylcarnitine	1.02	0.80↓*	1.18	0.94
	Octanoylcarnitine	0.97	0.76↓*	1.21	1.00
	Decanoylcarnitine	0.97	0.75↓*	1.39	1.08
	Myristoleoylcarnitine	1.00	0.75↓*	1.32	0.85
	Suberoylcarnitine	1.73↑*	0.98	4.08	3.07
	Arachidoylcarnitine	0.88	1.09	0.73↓**	1.06
	Arachidonoylcarnitine	1.14	1.11	1.31↑*	1.05
	Adrenoylcarnitine	1.06	1.10	1.30	1.13
	Behenoylcarnitine	0.81	1.18	0.65↓**	1.04
	Dihomo-linolenoylcarnitine	1.05	1.03	1.31↑***	1.05
	Lignoceroylcarnitine	0.84↓*	1.12	0.71↓**	0.90
	Margaroylcarnitine	1.01	0.84↓*	1.09	1.06
	Cerotoylcarnitine	0.91	1.11	0.89↓*	0.92

Carnitine metabolism	Deoxycarnitine	0.92↓*	0.93	1.04	1.00

Ketone bodies	Acetoacetate	2.08	1.50	2.13	0.67↓*

Fatty acid, monohydroxy	Alpha-hydroxycaproate	4.85	1.00	12.46↑**	0.94
	2-Hydroxypalmitate	0.85	0.82↓*	1.13	0.89
	3-Hydroxylaurate	0.78	0.74↓*	1.33	0.87
	3-Hydroxymyristate	0.91	0.77↓*	1.27	0.83
	16-Hydroxypalmitate	0.96	0.92	1.38↑*	0.98
	9-Hydroxystearate	1.09	0.72↓**	1.29	0.97

Fatty acid, dihydroxy	12,13-DiHOME	1.12	1.36↑*	1.18	0.88

Endocannabinoid	Oleoyl ethanolamide	0.90	0.77↓*	1.13	0.95
	Palmitoyl ethanolamide	0.98	0.81↓*	1.15	1.04
	Arachidonoyl ethanolamide	1.24	0.94	1.77↑*	1.18
	*N*-oleoyltaurine	0.49↓**	0.94	1.03	1.74
	*N*-palmitoyltaurine	0.56↓**	1.12	1.46	1.48

Phospholipid metabolism	Choline phosphate	1.97	2.72	0.65↓*	1.49
	1-Linoleoyl-2-linolenoyl-GPC	1.63	2.11↑*	1.22	1.10
	1-Palmitoyl-2-palmitoleoyl-GPC	1.08	0.98	1.30	1.48↑*
	Docosahexaenoylcholine	1.25	0.98	1.08	0.82↓*

Lysolipid	2-Palmitoleoyl-GPC	0.95	1.31	1.95↑*	2.65↑**
	1-Palmitoyl-GPG	0.83	0.90	1.35	2.05↑*
	1-Oleoyl-GPG	0.85	0.75↓*	0.84	2.12

Plasmalogen	1-(1-Enyl-stearoyl)-2-linoleoyl-GPE	1.25↑*	1.10	1.14	1.09

Monoacylglycerol	1-Pentadecanoylglycerol	0.71↓*	0.72↓*	1.02	1.10
	1-Palmitoylglycerol	0.76↓**	0.86	1.07	1.03
	2-Palmitoylglycerol	0.77↓*	0.79↓*	1.05	1.01
	1-Margaroylglycerol	0.80	0.85	1.46	0.63↓*
	1-Oleoylglycerol	0.85↓*	0.88	0.90	0.90
	2-Oleoylglycerol	0.81	0.92	0.80↓*	1.05
	1-Dihomo-linolenylglycerol	0.81↓*	0.77↓*	1.17	0.99

Diacylglycerol	Diacylglycerol (12:0/18:1, 14:0/16:1, 16:0/14:1) [2]	1.83	1.77	5.07	8.03↑**
	Diacylglycerol (14:0/18:1, 16:0/16:1) [1]	1.13	1.29	2.39	3.42↑*
	Diacylglycerol (14:0/18:1, 16:0/16:1) [2]	1.17	1.13	1.99	2.70↑*
	Linoleoyl-linolenoyl-glycerol (18:2/18:3) [1]	1.12	1.71	2.28↑**	0.93
	Palmitoyl-myristoyl-glycerol (16:0/14:0) [2]	2.14	1.36	2.50	3.84↑**
	Palmitoyl-palmitoyl-glycerol (16:0/16:0) [2]	1.08	0.91	1.88↑*	1.91
	Palmitoyl-linolenoyl-glycerol (16:0/18:3) [2]	1.10	1.35	4.44↑**	1.41

Sphingolipid metabolism	*N*-behenoyl-sphingadienine (d18:2/22:0)	0.92	0.99	0.91↓*	0.87↓**
	Myristoyl dihydrosphingomyelin (d18:0/14:0)	1.13	0.96	1.26↑*	1.01
	Sphingomyelin (d18:1/18:1, d18:2/18:0)	1.01	0.98	1.19↑*	0.97
	Sphingomyelin (d18:1/20:0, d16:1/22:0)	0.95	1.00	0.91↓*	0.93
	Sphingomyelin (d18:1/22:1, d18:2/22:0, d16:1/24:1)	0.98	0.98	0.91↓*	0.94
	*N*-palmitoyl-sphingosine (d18:1/16:0)	1.05	1.02	1.44↑*	1.05
	Glycosyl-N-palmitoyl-sphingosine (d18:1/16:0)	1.03	0.97	0.91↓*	0.94
	Sphingomyelin (d18:1/20:2, d18:2/20:1, d16:1/22:2)	1.22	1.02	1.55↑**	0.98
	Sphingomyelin (d18:2/24:2)	1.25	1.06	1.37↑*	1.02
	*N*-nervonoyl-hexadecasphingosine (d16:1/24:1)	1.09	0.91	1.22↑*	1.02
	Sphingomyelin (d18:1/22:2, d18:2/22:1, d16:1/24:2)	1.22	1.02	1.38↑*	0.99

Sterol	3beta-hydroxy-5-cholestenoate	0.87	0.84↓*	1.08	0.93

Steroid	Pregnenolone sulfate	0.85↓*	0.93	1.04	1.14
	21-Hydroxypregnenolone disulfate	0.99	1.08	1.52↑*	0.93
	5alpha-pregnan-3beta,20beta-diol monosulfate (1)	0.77↓*	0.94	1.09	1.05
	5alpha-pregnan-3beta,20alpha-diol monosulfate (2)	0.81↓*	0.89	0.96	1.10
	Pregn steroid monosulfate	0.77↓***	0.90	1.12	1.00
	Pregnanediol-3-glucuronide	0.74↓**	1.00	1.18	1.11
	16a-Hydroxy DHEA 3-sulfate	1.29	1.07	1.47↑**	1.06
	Androstenediol (3beta,17beta) monosulfate (2)	1.12	1.04	1.20↑*	0.94
	Androstenediol (3beta,17beta) disulfate (1)	1.13	1.01	1.21↑*	0.95
	Androstenediol (3beta,17beta) disulfate (2)	1.05	1.00	1.26↑**	0.99
	5alpha-androstan-3alpha,17beta-diol monosulfate (1)	1.08	0.93	1.43↑**	1.02
	5alpha-androstan-3alpha,17beta-diol disulfate	0.73↓*	1.18	1.04	0.80
	Andro steroid monosulfate (1)	1.52	1.01	1.54↑*	1.18

Primary bile acid metabolism	Taurocholate	4.56	1.24	3.65↑*	2.30
	Taurochenodeoxycholate	6.55	1.25	2.58	2.81↑*
	Tauro-beta-muricholate	2.42	1.12	2.29↑*	2.16
	Glycocholate glucuronide (1)	1.00	1.01	1.00	0.93↓*

Secondary bile acid metabolism	Glycodeoxycholate	0.97	4.27↑*	1.10↑*	1.37
	Glycohyocholate	3.75↑*	1.29	1.94	1.55
	7-Ketodeoxycholate	0.55↓**	1.26	1.01	1.04

Ceramides	Ceramide (d18:1/14:0, d16:1/16:0)	1.09	0.93	1.22↑*	1.07
	Ceramide (d18:1/20:0, d16:1/22:0, d20:1/18:0)	0.95	1.01	0.97	0.90↓*
	Ceramide (d18:2/24:1, d18:1/24:2)	1.04	0.93	1.16	0.87↓*

Fatty acid metabolism (acyl choline)	Linoleoylcholine	6.95	1.75	4.17	0.63↓*

*DHEA, dehydroepiandrosterone; 12,13-DiHOME, 12,13-dihydroxy-9Z-octadecenoic acid; GPC, glycerophosphocholine; GPE, glycerophosphoethanolamine; GPG, glycerophosphoglycerol*.

*^a^Metabolites that were detected in plasma but non-significant (*p* > 0.05) in dietary groups compared to respective baseline are not presented in this table*.

^b^Values presented are mean metabolite ratios/fold changes. Statistically significantly increased (↑) fold changes are bolded and highlighted in red, and statistically significantly decreased (↓) fold changes are bolded and highlighted in blue (*p ≤ 0.05; **p ≤ 0.01; ***p ≤ 0.001).

### Differences in Plasma Amino Acid Metabolites following Navy Bean and/or Rice Bran Consumption

As indicated in Figure 2, aside from lipids, metabolites within the amino acid class were also significantly modulated by navy bean and/or rice bran consumption (Table 3; Figures S1A,C,E in Supplementary Material). Compared to control at 4 weeks, navy bean consumption decreased seven amino acid metabolites, including *O*-acetylhomoserine, phenylalanine, isovalerylcarnitine, 3-hydroxyisobutyrate, methionine, methionine sulfoxide, and oxidized cysteine-glycine dipeptide (0.53- to 0.84-fold difference), and increased 6-oxopiperidine-2-carboxylate (1.87-fold difference) (Table 3). Rice bran consumption decreased seven amino acids compared to control at 4 weeks, including three histidine metabolites (0.28- to 0.57-fold difference), isovalerylglycine, isovalerylcarnitine, 5-hydroxyindoleacetate, and *N*-formylmethionine (Table 3). The consumption of navy bean + rice bran significantly modulated four amino acid metabolites compared to control, with a 3.11-fold difference increase in gentisate, and significant decreases in betaine, 4-methyl-2-oxopentanoate, and 3-methyl-2-oxovalerate (Table 3).

**Table 3:** Other modulated plasma metabolites following navy bean and/or rice bran consumption for 4 weeks compared to control.^a^

Metabolic pathway	Metabolite	Fold difference vs. control^b^		
		Navy bean	Rice bran	Navy bean + Rice bran
**Amino acids**				
Gly, Ser, and Thr metabolism	Betaine	0.93	0.93	0.85↓**
	*O*-acetylhomoserine	0.53↓*	0.66	1.58
His metabolism	3-Methylhistidine	0.87	0.28↓*	0.39
	*N*-acetyl-1-methylhistidine	0.96	0.53↓*	0.72
	Trans-urocanate	0.91	0.57↓**	0.99
Lys metabolism	6-Oxopiperidine-2-carboxylate	1.87↑**	1.49	1.49
Phe and Tyr metabolism	Phenylalanine	0.86↓**	1.00	0.95
	Gentisate	1.96	1.48	3.11↑*
Trp metabolism	5-Hydroxyindoleacetate	0.67	0.67↓*	0.98
Leu, Ile, and Val metabolism	4-Methyl-2-oxopentanoate	0.96	0.96	0.79↓*
	Isovalerylglycine	0.68	0.34↓**	0.54
	Isovalerylcarnitine	0.67↓*	0.62↓**	0.72
	3-Methyl-2-oxovalerate	0.97	0.94	0.78↓*
	3-Hydroxyisobutyrate	0.61↓*	1.01	0.74
Met, Cys, SAM, and Taurine metabolism	Methionine	0.84↓**	0.91	0.95
	*N*-formylmethionine	0.99	0.85↓**	1.07
	Methionine sulfoxide	0.77↓*	0.86	0.86
Glutathione metabolism	cys-gly, oxidized	0.81↓*	0.74	0.99

**Peptide**				
Gamma-glutamyl amino acid	Gamma-glutamyl-epsilon-lysine	1.25	1.30	1.37↑*

**Carbohydrate**				
Pentose metabolism	Ribitol	0.79↓*	0.85	0.78↓*

**Nucleotide**				
Purine metabolism, (hypo)xanthine/inosine	Urate	0.89	0.84	0.83↓*
Purine metabolism, adenine	Adenosine	2.19	0.09↓*	0.23
Pyrimidine metabolism, cytidine	Cytidine	1.20	0.35↓*	0.77

**Cofactors/vitamins**				
Nicotinate and nicotinamide metabolism	1-Methylnicotinamide	0.58↓**	0.79	1.03
	Trigonelline (*N*’-methylnicotinate)	4.44↑*	1.51	1.39
Hemoglobin and porphyrin metabolism	Heme	0.79	0.56↓**	0.89
Vitamin B6 metabolism	Pyridoxal	1.47	3.35↑**	1.63

**Phytochemicals/other**				
Benzoate metabolism	3-Methyl catechol sulfate	1.82	0.43	0.36↓*
	4-Vinylphenol sulfate	2.22↑*	0.57	1.62
Food component/plant	2-Isopropylmalate	1.01	0.79↓*	0.98
	*N*-acetylalliin	0.51↓*	0.72	0.53
	Erythritol	0.85↓*	0.92	0.93
	Saccharin	0.96	0.21↓**	0.49
	Theanine	1.00	1.00	8.11↑*
Drug	Omeprazole	0.47↓*	0.47↓*	0.47↓*
Chemical	*N*-methylpipecolate	1.89↑*	1.94	0.98

*Cys, cysteine; Gly, glycine; His, histidine; Ile, isoleucine; Leu, leucine; Lys, lysine; Met, methionine; Phe, phenylalanine; SAM, S-adenosyl methionine; Ser, serine; Thr, threonine; Trp, tryptophan; Tyr, tyrosine; Val, valine*.

*^a^Metabolites that were detected in plasma but non-significant (*p* > 0.05) in dietary groups compared to control are not presented in this table*.

^b^Values presented are mean metabolite ratios/fold differences. Statistically significantly increased (↑) fold differences are bolded and highlighted in red, and statistically significantly decreased (↓) fold differences are bolded and highlighted in blue (*p ≤ 0.05; **p ≤ 0.01).

Compared to baseline, navy bean consumption for 4 weeks increased seven amino acids, including pipecolate (2.86-fold change), *S*-methylcysteine (2.12-fold change), and *S*-methylcysteine sulfoxide (2.19-fold change), along with 2,3-dihydroxy-2-methylbutyrate, 2-hydroxy-3-methylvalerate, *N*-acetyl-3-methylhistidine, and *N*-delta-acetylornithine. Five amino acids were decreased following navy bean consumption compared to baseline (*N*-acetylglycine, kynurenate, methylsuccinylcholine, *N*-alpha-acetylornithine, *N*-acetylcitrulline) (Table S1 in Supplementary Material; Figures S1B,D,F in Supplementary Material). Rice bran consumption for 4 weeks compared to baseline resulted in an increase in 24 amino acids across multiple metabolic pathways, among which included 5 phenylalanine and tyrosine metabolites (1.11- to 2.32-fold change), 3 urea cycle metabolites (1.09- to 1.23-fold change), and 3 methionine and cysteine metabolites (1.20- to 3.34-fold change). The navy bean + rice bran combination group had limited effects on amino acid metabolites compared to baseline (1 increased and 1 decreased). Interestingly, the control group also increased 6 and decreased 2 amino acids.

### Phytochemicals Detected and Modulated following Navy Bean and/or Rice Bran Consumption

Phytochemicals (and other exogenous compounds) represented the third largest metabolic grouping modulated by navy bean and/or rice bran consumption (Figure 2). Compared to control, navy bean consumption increased the phytochemicals 4-vinylphenol sulfate (2.22-fold difference) and *N*-methylpipecolate (1.89-fold difference), and decreased *N*-acetylalliin, erythritol, and the drug compound omeprazole (Table 2; Figures 2A,C,E in Supplementary Material). Rice bran consumption decreased three phytochemicals and exogenous chemicals compared to control, including 2-isopropylmalate, saccharin, and omeprazole. Navy bean + rice bran consumption increased theanine (8.11-fold difference) and decreased 3-methyl catechol sulfate and omeprazole, compared to control.

Compared to baseline, navy bean consumption for 4 weeks decreased 8 phytochemicals and exogenous compounds, including 7 xanthine metabolites (0.50- to 0.79-fold change), and 4-hydroxylchlorothalonil (0.92-fold change) (Table S1 in Supplementary Material; Figures S2B,D,F in Supplementary Material). Several phytochemicals and exogenous compounds were also increased in the navy bean group compared to baseline with large magnitude fold changes, including ferulic acid 4-sulfate (4.62-fold change), 4-vinylguaiacol sulfate (20.17-fold change), dimethyl sulfone (1.54-fold change), and ectoine (23.49-fold change). Phytochemicals and exogenous compounds increased following rice bran consumption compared to baseline included the benzoate metabolite 2-hydroxyhippurate (3.05-fold change), alliin (3.34-fold change), salicylate (2.74-fold change), *S*-carboxymethyl-l-cysteine (3.14-fold change), 2-keto-3-deoxy-gluconate, *N*-methylpipecolate, and 4-hydroxychlorothalonil. One food metabolite, stachydrine, was decreased in rice bran group. Ten phytochemicals and exogenous compounds were increased in the navy bean + rice bran group compared to baseline, including seven xanthine metabolites (2.50- to 10.85-fold change), allylphenol sulfate (4.47-fold change), methyl glucopyranoside (2.55-fold change), and dimethyl sulfone (1.72-fold change), and two phytochemicals were decreased (*N*-acetylalliin and 3,4-methyleneheptanoate). Phytochemicals and exogenous compounds increased in the control group include 4-allylphenol sulfate (9.19-fold change), umbelliferone sulfate (6.74-fold change), paraxanthine (2.65-fold change), 5-acetylamino-6-amino-3-methyluracil (7.28-fold change), *N*-acetylalliin (1.98-fold change), and omeprazole (2.14-fold change), and decreased phytochemicals include cotinine, retinal, and *N*-methylpipecolate.

### Other Metabolic Pathways Modulated by Navy Bean and/or Rice Bran Consumption

In comparison to lipids, amino acids, and phytochemicals, fewer metabolites from the cofactor/vitamin, carbohydrate, peptide, nucleotide, energy pathways were significantly modulated by navy bean and/or rice bran consumption (Figure 2). Yet, large magnitude fold changes/differences from these pathways are of possible health importance and may also represent novel dietary biomarkers of intake. Navy bean consumption compared to control significantly increased the vitamin B3 metabolite trigonelline (4.44-fold difference), and decreased the relative abundances of the carbohydrate metabolite ribitol and the nicotinamide metabolite 1-methylnicotinamide (Table 2). In the rice bran group, a large 3.35-fold difference increase in pyridoxal (vitamin B6) was observed compared to control at 4 weeks. Several metabolites from the nucleotide and cofactor/vitamin metabolic pathways were decreased following rice bran consumption compared to control, including adenoside, cytidine, and heme. In the navy bean + rice bran group, an increase in the peptide gamma-glutamyl amino acid, and decreases in ribitol, urate, 3-methyl catechol sulfate, and omeprazole, were determined.

Compared to baseline, a significant 7.65-fold change increase in trigonelline was also observed in the navy bean group (Table 2). Navy bean consumption also decreased the hemoglobin metabolite bilirubin (E,E isomeric forms) (0.69-fold change) compared to baseline, but had no to limited modulatory effects on carbohydrate, nucleotide, peptide, or energy pathway metabolites. Rice bran consumption increased 4 cofactors/vitamins compared to baseline, including a 3.96-fold change increase in pyridoxal (vitamin B6), as well as bilirubin (Z, Z; E, Z; and Z, E isomeric forms), and pantothenate. In addition, six nucleotides were increased by rice bran consumption, with the largest fold changes observed for hypoxanthine (3.15-fold change) and dihydroorotate (2.58-fold change), while two nucleotides were decreased (0.65- to 0.71-fold change). Rice bran consumption compared to baseline also increased 2 (1.17- to 1.18-fold change) and decreased 4 (0.41- to 0.91-fold change) carbohydrates, and nine peptides (gamma-glutamyl amino acids; 1.21- to 2.27-fold change). However, limited effects were observed on energy metabolites following rice bran consumption compared to baseline. Consumption of navy bean + rice bran for 4 weeks compared to baseline increased ascorbate (vitamin C) (1.99-fold change), and decreased the tocopherol metabolite alpha-CEHC sulfate (0.70-fold change). Three peptide metabolites were also increased by navy bean + rice bran consumption (gamma-glutamyltyrosine, fibrinogen cleavage peptide, and phenylacetylglutamate); however, there were limited modulatory effects on carbohydrate, nucleotide, and energy pathway metabolites. The control group modulated four nucleotides (2 increased, 2 decreased), decreased 2 peptides, and decreased the hemoglobin metabolite bilirubin (E, E isomer) compared to baseline, but had no effects on carbohydrates, energy pathway metabolites.

### Plasma Metabolites Detected in the Food Metabolome

Of the 854 metabolites identified in plasma, 321 were also detected in the food metabolome in a parent or intermediate form (267 from navy bean, 300 from rice bran) (Table S2 in Supplementary Material). For navy bean, food metabolites detected with high relative abundance in plasma (fold change/difference > 2) included trigonelline (4.44-fold difference; 7.65-fold change), ferulic acid 4-sulfate (4.62-fold change), pipecolate (2.86-fold change), and *S*-methylcysteine (2.12-fold change), and *S*-methylcysteine sulfoxide (2.19-fold change). Rice bran metabolites detected with high relative abundance in plasma include methionine sulfone (3.34-fold change), alpha-hydroxycaproate (12.46-fold change), linoleoyl-linolenoyl-glycerol (2.28-fold change), palmitoyl-linolenoyl-glycerol (4.44-fold change), pyridoxal (3.35-fold difference; 3.96-fold change), 2-hydroxyhippurate (3.05-fold change), salicylate (2.74-fold change), gamma-glutamylglutamate (2.17-fold change), gamma-glutamylthreonine (2.27-fold change), hypoxanthine (3.15-fold change), and dihydroorotate (2.58-fold change).

## Discussion

Using a non-targeted metabolomics approach, we demonstrated that navy bean and/or rice bran consumption for 4 weeks significantly modulated the plasma metabolome of children with abnormal cholesterol. Lipid metabolites represented 46% of total metabolites identified, and significant changes determined for 18 plasma lipids in the navy bean group and 10 plasma lipids for the rice bran group compared to control, and 48 lipids in the navy bean group and 40 in the rice bran group compared to baseline, support the hypothesis that consumption of these foods impact blood lipid metabolism with implications for reducing CVD risk in children. The findings in this paper advance upon previous efforts, whereby navy beans and/or rice bran demonstrated total and LDL-cholesterol-lowering properties (31, 32) as well as increasing HDL-cholesterol in children, adults, and/or experimental animals (19, 23–27).

### Modulations in Plasma Lipid Metabolites and CVD Risk

Distinct subgroups of lipid metabolic pathways were differentially altered by consumption of navy bean and/or rice bran. Compared to the control group, navy bean consumption increased several acylcarnitines, lysolipids, and ceramides, whereas rice bran consumption increased sphingolipids and ceramides, and navy bean + rice bran consumption increased lysolipids and phospholipids. Increased phospholipids were also determined for navy bean or rice bran consumption alone (1-palmitoleoyl-2-linolenoyl-glycerophosphocholine in the navy bean group, and 1-palmitoyl-2-oleoyl-glycerophosphocholine in the rice bran group), with high complementary pathway enrichment (score = 4.9) for 3 phospholipids in the navy bean + rice bran group. Lysolipids, sphingolipids, and phospholipids are critical components of cell membranes and important for many cell signaling processes that impact cholesterol absorption and bile acid secretion (33–35). Carnitines/acylcarnitines and ceramides are essential compounds for fatty acid metabolism and β-oxidation, and are derived from animal, plant, and/or microbial sources (36–38). However, increased acylcarnitines may also reflect inherited disorders in fatty acid and branched-chain amino acid catabolism associated with CVD, and increased ceramides can impact atherosclerosis and inflammation (36–38). The dual-functions of these lipids make it difficult to discern the biological significance of their relative increase in plasma, and whether the net change is beneficial long term. Additionally, since acylcarnitines are present in many foods at varying abundances, it is plausible that incorporation of navy beans and/or rice bran in the diet displaces animal-based foods that may be higher in saturated fats and cholesterol (39). Compared to baseline, navy bean consumption decreased multiple long-chain fatty acids and polyunsaturated fatty acids. Palmitate, a saturated long-chain fatty acid found from both animal and plant sources, has been associated with insulin resistance and an increased risk of CVD (38). Additionally, arachidonate, a polyunsaturated omega-6 fatty acid, is a precursor for many pro-inflammatory prostaglandins and leukotrienes and decreased plasma levels following navy bean consumption may reduce inflammatory signally associated with metabolic risk (40).

Furthermore, modulations in several endogenous lipids are noteworthy for cardioprotection. Endocannabinoids, a group of endogenous bioactive lipids, were decreased in 4-week navy bean plasma compared to baseline. Activation of cannabinoid (CB1) receptors by endocannabinoids has been implicated in the development of various cardiovascular disorders, including obesity, metabolic syndrome, insulin resistance, diabetes, and general inflammation (41). Further, *N*-linoleoylglycine, an endogenous compound increased following navy bean consumption, promotes the synthesis of anti-inflammatory eicosanoids, such as 15-deoxy-Δ^13,14^-prostaglandin J2 and lipoxin A_4_, that protect against chronic inflammation (42). Given that chronic inflammation is intricately linked to CVD progression (43), investigation into how navy beans can reduce systemic inflammation could support their utility in chronic disease control. Navy bean consumption compared to baseline also increased the dihydroxy fatty acid metabolite 12,13-dihydroxy-9Z-octadecenoic acid (12,13-diHOME), an endogenous lipokine increased from brown adipose tissue following exposure to cold temperatures, that been purported to be beneficial for metabolic disorders including obesity, diabetes, and hyperlipidemia (44). Additionally, significant increases in several primary and secondary bile acid metabolites following navy bean and/or rice bran consumption (compared to control and/or baseline) suggest dietary promotion of lipid solubilization/absorption and biliary lipid secretion, which could be beneficial for growth in infants and young children, and warrants further investigation (45).

### Navy Bean and Rice Bran-Derived Metabolites and CVD Risk

Navy bean and/or rice bran consumption also increased multiple amino acids, phytochemicals, and cofactors/vitamins of dietary origin with nutritional and health importance. Navy beans are a rich source of the essential amino acid lysine, and several lysine-derived amino acids and phytochemicals (6-oxopiperidine-2-carboxylate, pipecolate, *N*-methylpipecolate) were increased in plasma following navy bean consumption (19). Along with boosting nutritional status, lysine acts as a precursor in the synthesis of branched-chain fatty acids that are inversely associated CVD risk (36, 46). Multiple ferulic acid metabolites were also increased following navy bean intake compared to baseline, including ferulic acid 4-sulfate and 4-vinylguaiacol sulfate. Ferulic acid is a phytochemical with strong lipid antioxidant properties, and scavenges free radicals to prevent lipid peroxidation (47). Navy bean consumption compared to control and baseline also increased *S*-methylcysteine, along with its oxidized product, *S*-methylcysteine sulfoxide, both of which have antioxidant and antidiabetic functions with implications to attenuate inflammation and metabolic diseases (48, 49). Furthermore, the B-vitamin metabolite trigonelline increased 4.44-fold following navy bean consumption compared to control at 4 weeks (also increased 7.65-fold in navy bean compared to baseline). Trigonelline may contribute to reducing levels of blood lipids and preventing type 2 diabetes, through inhibiting lipase enzymes in the small intestine (50, 51).

For rice bran consumption, a 3.35-fold increase in pyridoxal (vitamin B6) compared to control (and 3.96-fold compared to baseline) is considered beneficial, as pyridoxal inhibits lipid glycation processes that typically occur in atherosclerosis, and also plays a role in converting homocysteine (a CVD risk marker) into methionine (52, 53). The observed increase in plasma pyridoxal boosts our previous findings of improved vitamin B6 nutritional status reported for children that received rice bran compared to groups that did not receive rice bran (27). In addition, increased pyridoxal following rice bran consumption has been observed in adults as well (29, 54). Rice bran consumption also increased the relative abundance of salicylate 2.74-fold compared to baseline. Salicylate, a phytochemical that plays a key function in plant defense systems, is also the active compound in aspirin, which has been demonstrated to reduce risk of heart diseases (55). The increased relative abundance of diet-derived bioactive metabolites in plasma following consumption of these practical and cost-effective foods offers a mechanistic explanation for navy bean and/or rice bran in cholesterol-lowering and providing nutritional support in children, as well as providing a sustainable alternative to pharmaceutical prevention of CVD. Further, these exogenous compounds may represent dietary biomarkers for navy bean and rice bran that will aid in completing larger cohort and epidemiological investigations.

### Study Limitations

Limitations to this study include the pilot trial aspects, such as a small cohort size and short study duration. The sample size (*n* = 38) is comparable though to other nutritional metabolomics-based human studies (*n* = 11 to 30 total participants) (29, 56–58). The tendency to select 1 or 2 compounds for future sample size estimates and power calculations warrants caution as this dietary intervention showed 10 to 20 metabolites differentially affected in growing children that could be of equal importance (59). Further, the complexity in the metabolite profiles affected by diet as detected *via* this sensitive, non-targeted metabolomics approach was with small doses of navy bean and/or rice bran (ranging 1.7 to 5.2% of the total caloric intake) (27), which indicates that small amounts of certain foods can be practically incorporated into the daily diets of children to influence underlying metabolic processes for CVD prevention. Further, we speculated that the fewer number of significant metabolites observed after 4 weeks of navy bean + rice bran consumption was dose-related. Investigations with larger daily intake doses of navy bean + rice bran as a combination warrants continued research attention to observe synergistic and complementary effects on the plasma metabolome.

## Conclusion

Incorporation of navy beans and rice bran in the diet has great potential to improve the health of children at risk for CVD. This study utilized a non-targeted metabolomics approach to reveal a wide spectrum of plasma lipids, amino acids, and phytochemicals modulated by navy bean and/or rice bran consumption, all of which are of significance to improving lipid profiles and overall nutritional status of children with early indices of dyslipidemia. Mitigating risk factors for CVD in children merits investigation with practical lifestyle interventions and this nutritional metabolomics approach offers great utility for the identification of dietary biomarkers that may further link navy bean and rice bran intake to long-term health benefits.

## Ethics Statement

This study was carried out in accordance with the recommendations of local and national guidelines and study protocols were approved by the University of Colorado Health-North Institutional Review Board (Protocol 13-1263) and the Colorado State University Research Integrity and Compliance Review Office (Protocol 13-4390). Prior to the start of the trial, written informed consent were obtained from guardians and written informed assent from all participants in accordance with the Declaration of Helsinki. This trial was registered at clinicaltrials.gov under NCT01911390.

## Author Contributions

KL carried out the metabolomics analyses and interpretation, drafted the initial manuscript, and reviewed and revised the manuscript. EB designed the study, supervised data collection, and reviewed and revised the manuscript. NJ-P and GL conceptualized and designed the study, coordinated and supervised data collection, and critically reviewed the manuscript. ER conceptualized and designed the study, supervised and performed metabolite profile results interpretations, and assisted in writing of the manuscript. All authors approved the final manuscript for submission.

## Conflict of Interest Statement

The authors declare that the research was conducted in the absence of any commercial or financial relationships that could be construed as a potential conflict of interest. The reviewer MS and handling editor declared their shared affiliation.

## Abbreviations

CVD, cardiovascular disease; GC-MS, gas-chromatography mass spectrometry; HDL, high-density lipoprotein; HILIC, hydrophilic interaction liquid chromatography; LDL, low-density lipoprotein; UPLC-MS/MS, ultra-high performance liquid-chromatography tandem mass spectrometry.
