# Supplementary material for: Navy Bean and Rice Bran Intake Alters the Plasma Metabolome of Children at Risk for Cardiovascular Disease
Source: Front Nutr. 2018 Jan 19;4:71. doi: 10.3389/fnut.2017.00071 (PMC5786740; doi:10.3389/fnut.2017.00071)
Supplement: Supplementary file 1 [file Data_Sheet_1.docx]

Supplementary Material

Navy Bean and Rice Bran Intake Alters the Plasma Metabolome of Children at Risk for Cardiovascular Disease

Katherine J. Li, Erica C. Borresen, NaNet Jenkins-Puccetti, Gary Luckasen, Elizabeth P. Ryan^*^

*** Correspondence:** Elizabeth P. Ryan: e.p.ryan@colostate.edu

# Supplementary Figures and Tables

## Supplementary Figures

**Supplemental Figure 1. Cytoscape pathway visualizations of amino acid metabolites modulated by Navy Bean, Rice Bran, or Navy Bean + Rice Bran consumption.** Amino acid metabolites modulated by Navy Bean, Rice Bran, or Navy Bean + Rice Bran consumption for 4 weeks compared to control (a,c,e) or compared to respective baseline (b,d,f), respectively. Nodes in red and blue represent significantly increased and decreased metabolites, respectively, compared to control at 4 weeks or compared to baseline. Node diameters are proportional to the magnitude of the fold-difference. Numbers within nodes represent pathway enrichment scores.

**(a)**

**
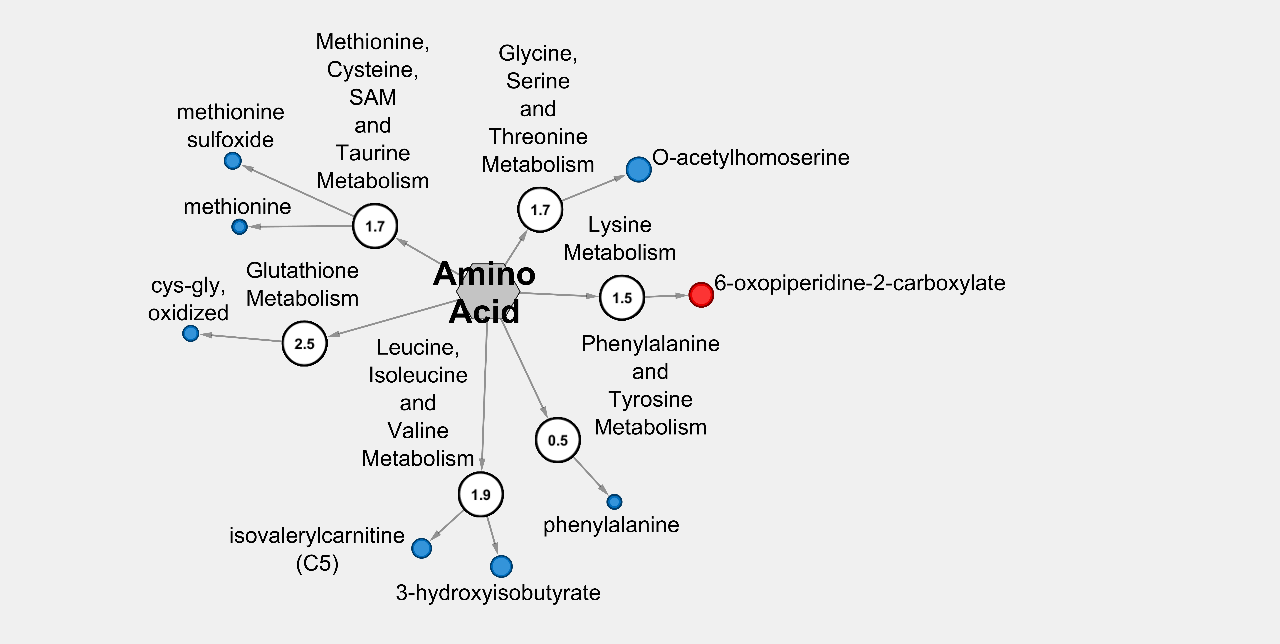
**

**(b)**

**
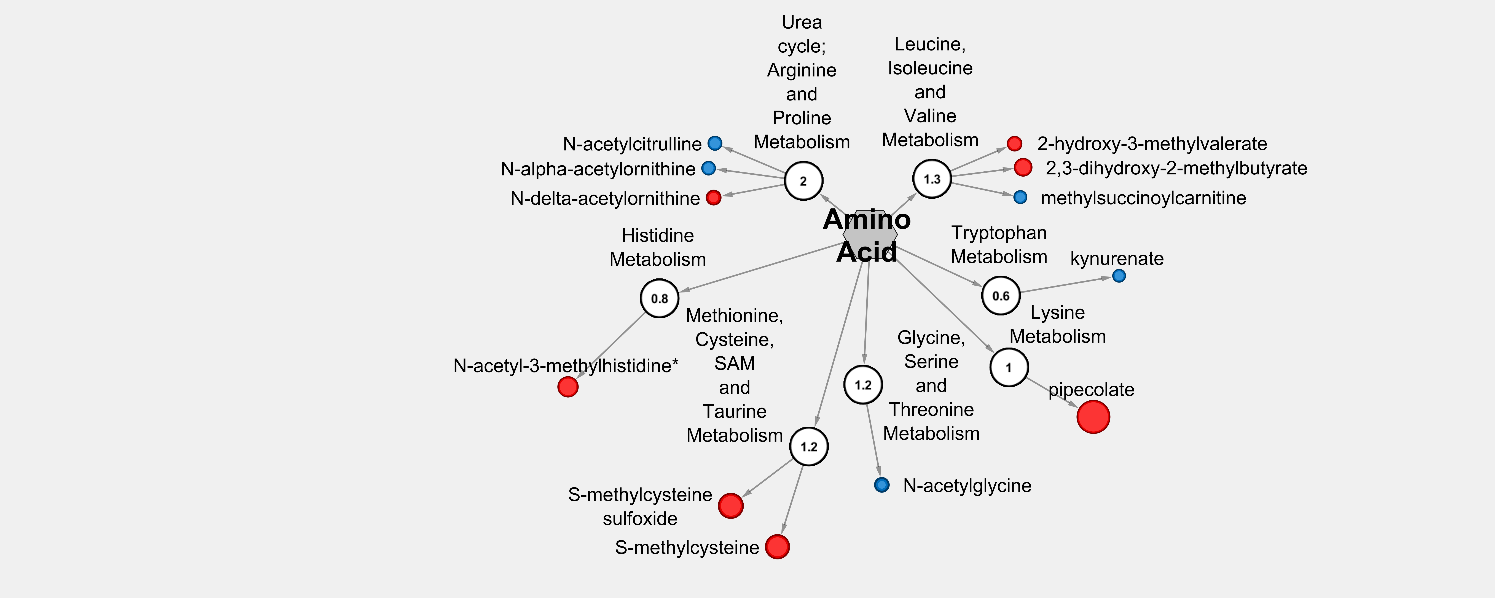
**

**(c)**

**
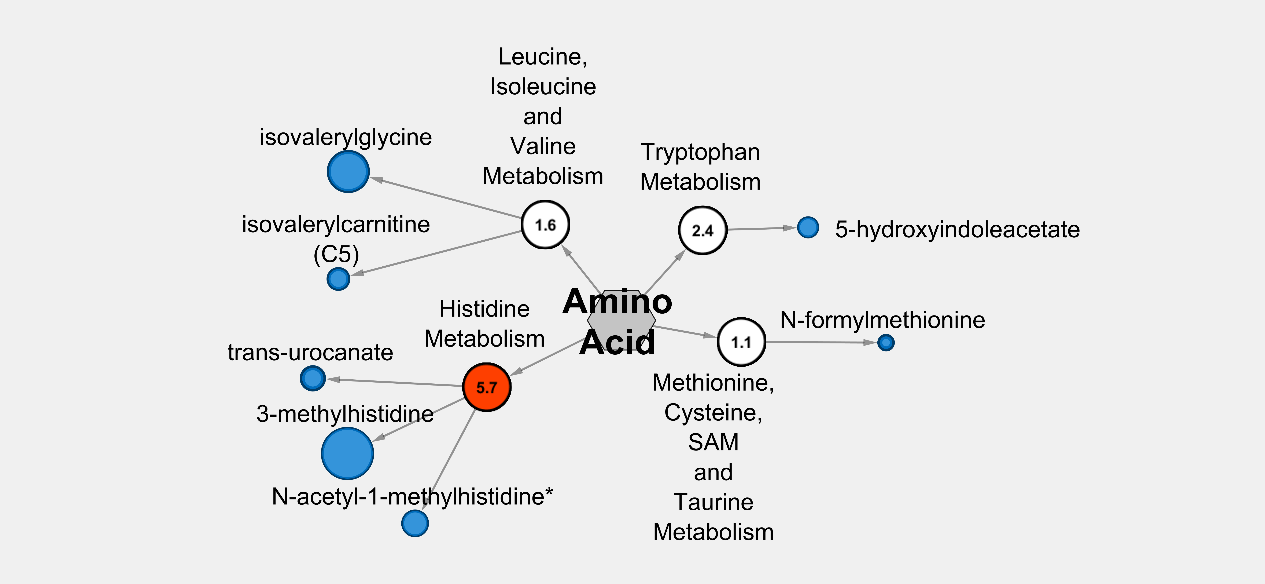
**

**(d)**

**
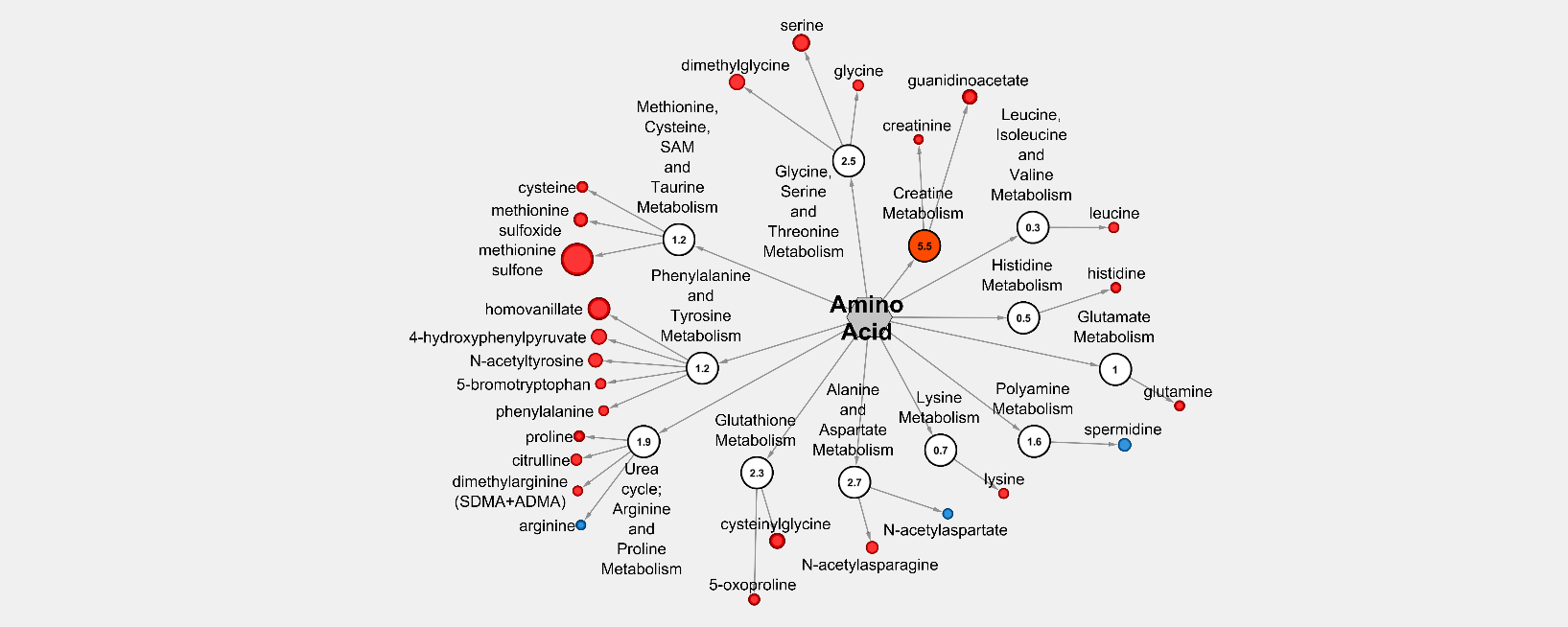
**

**(e)**

**
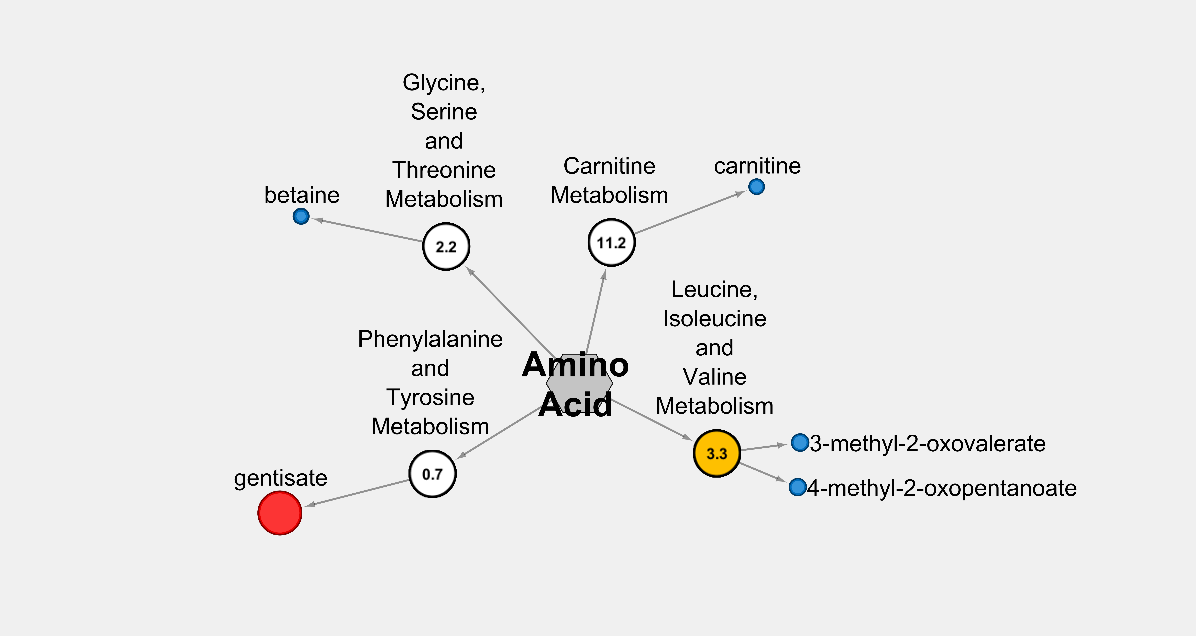
**

**(f)**

**
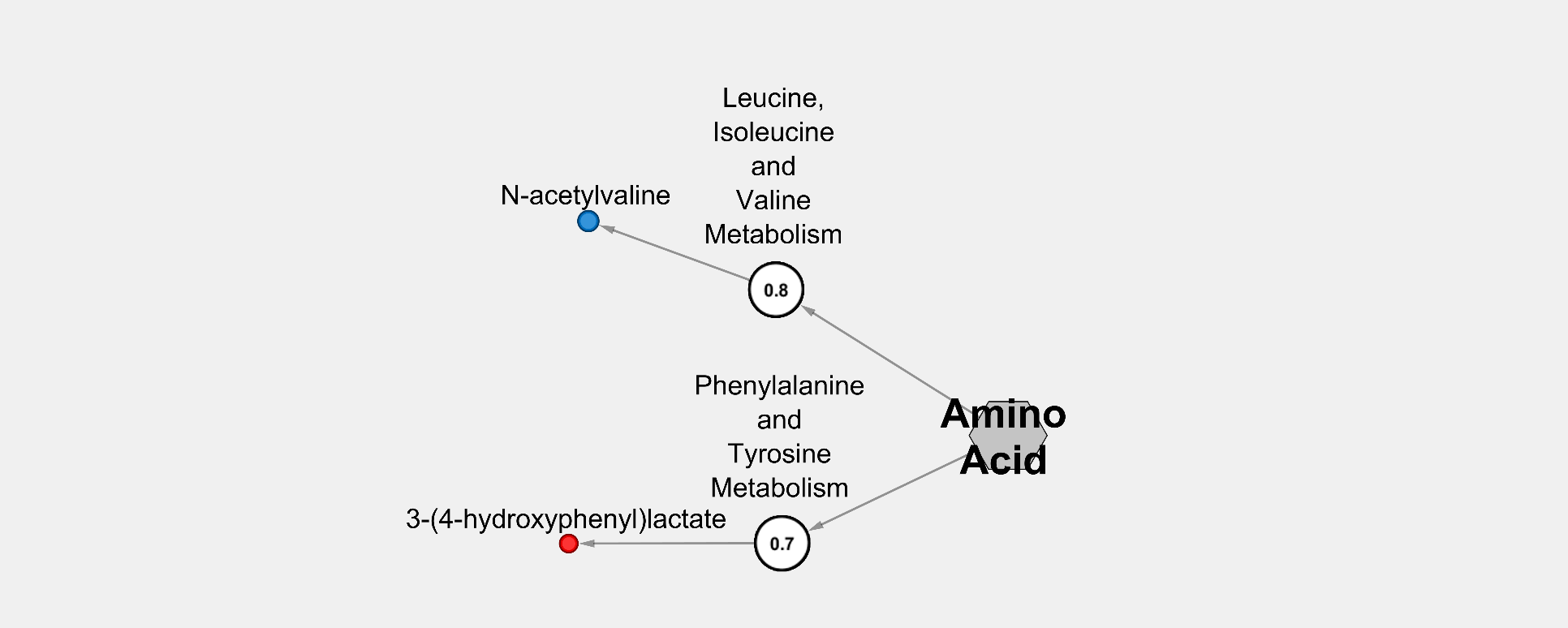
**

**Supplemental Figure 2. Cytoscape pathway visualizations of phytochemicals and exogenous metabolites metabolites modulated by Navy Bean, Rice Bran, or Navy Bean + Rice Bran consumption.** Phytochemicals and exogenous metabolites modulated by Navy Bean, Rice Bran, or Navy Bean + Rice Bran consumption for 4 weeks compared to control (a,c,e) or compared to respective baseline (b,d,f), respectively. Nodes in red and blue represent significantly increased and decreased metabolites, respectively, compared to control at 4 weeks or compared to baseline. Node diameters are proportional to the magnitude of the fold-difference. Numbers within nodes represent pathway enrichment scores.

**(a)**

**
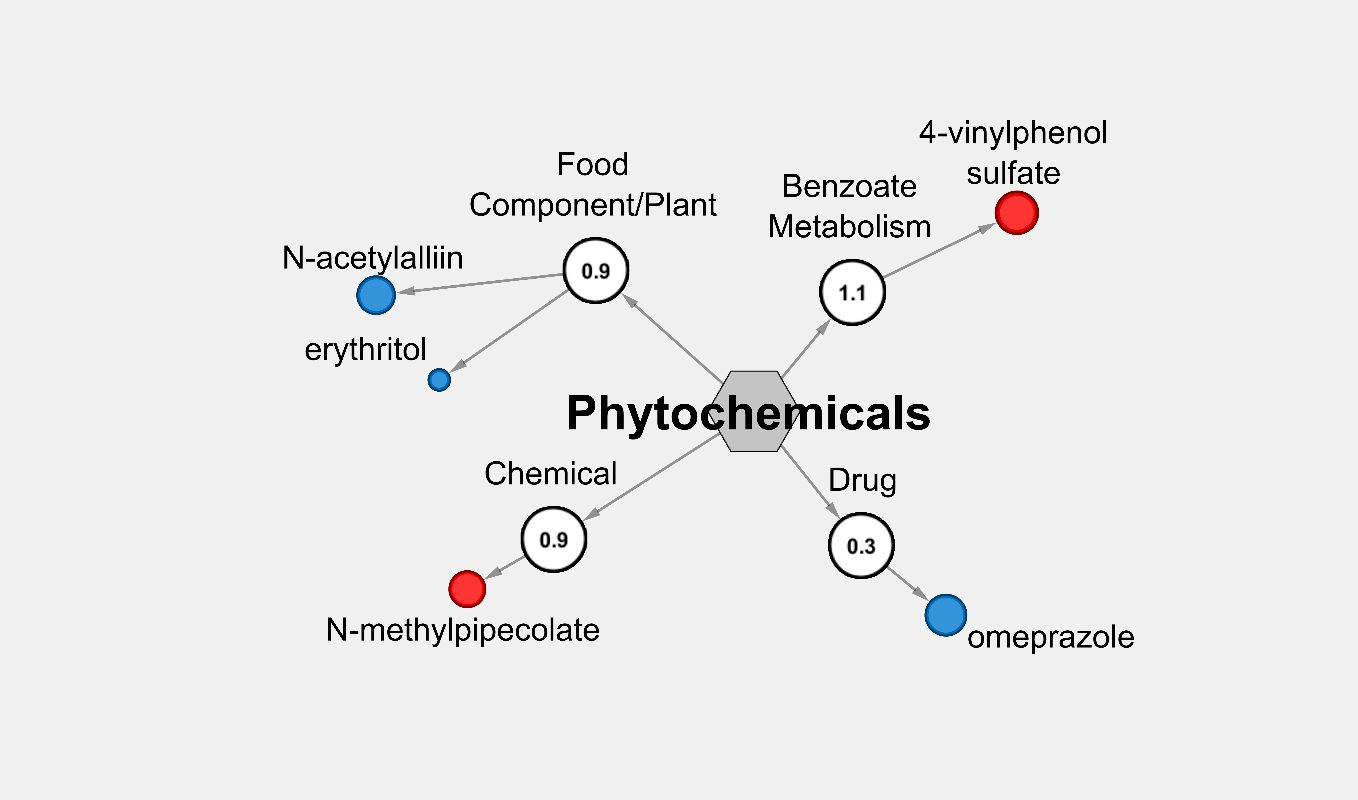
**

**(b)**

**
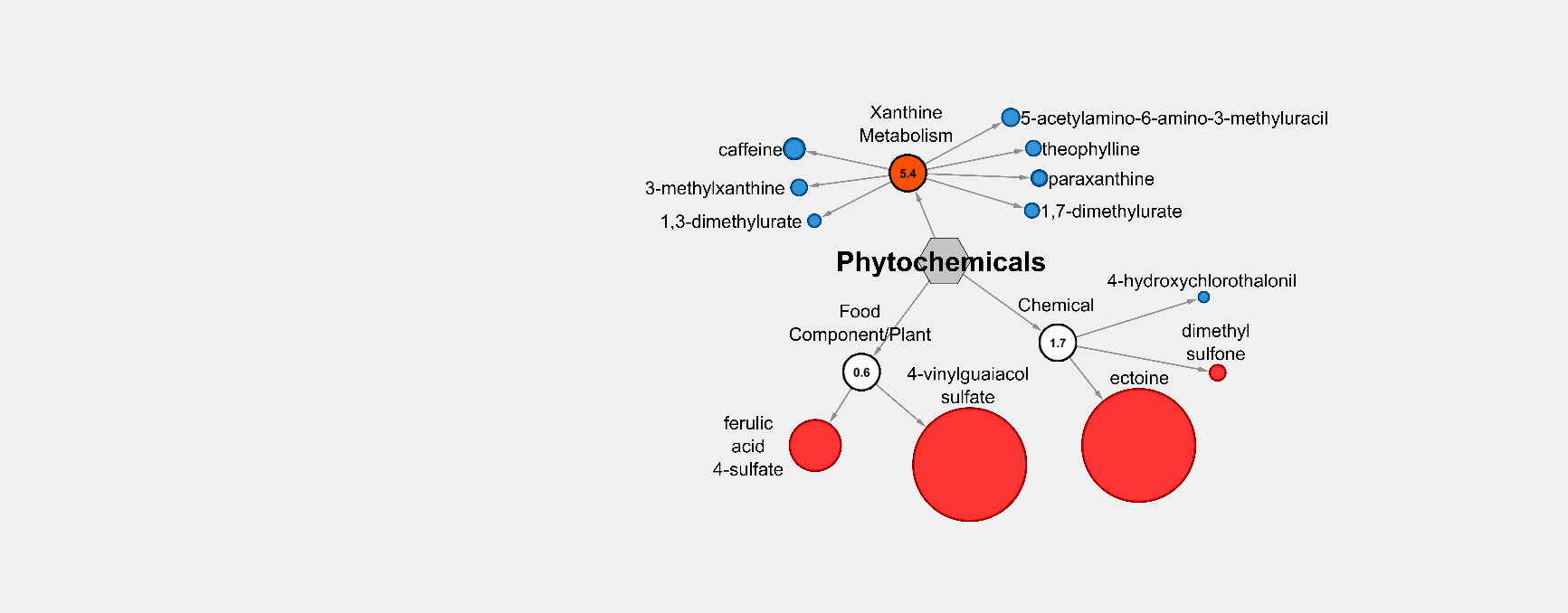
**

**(c)**

**
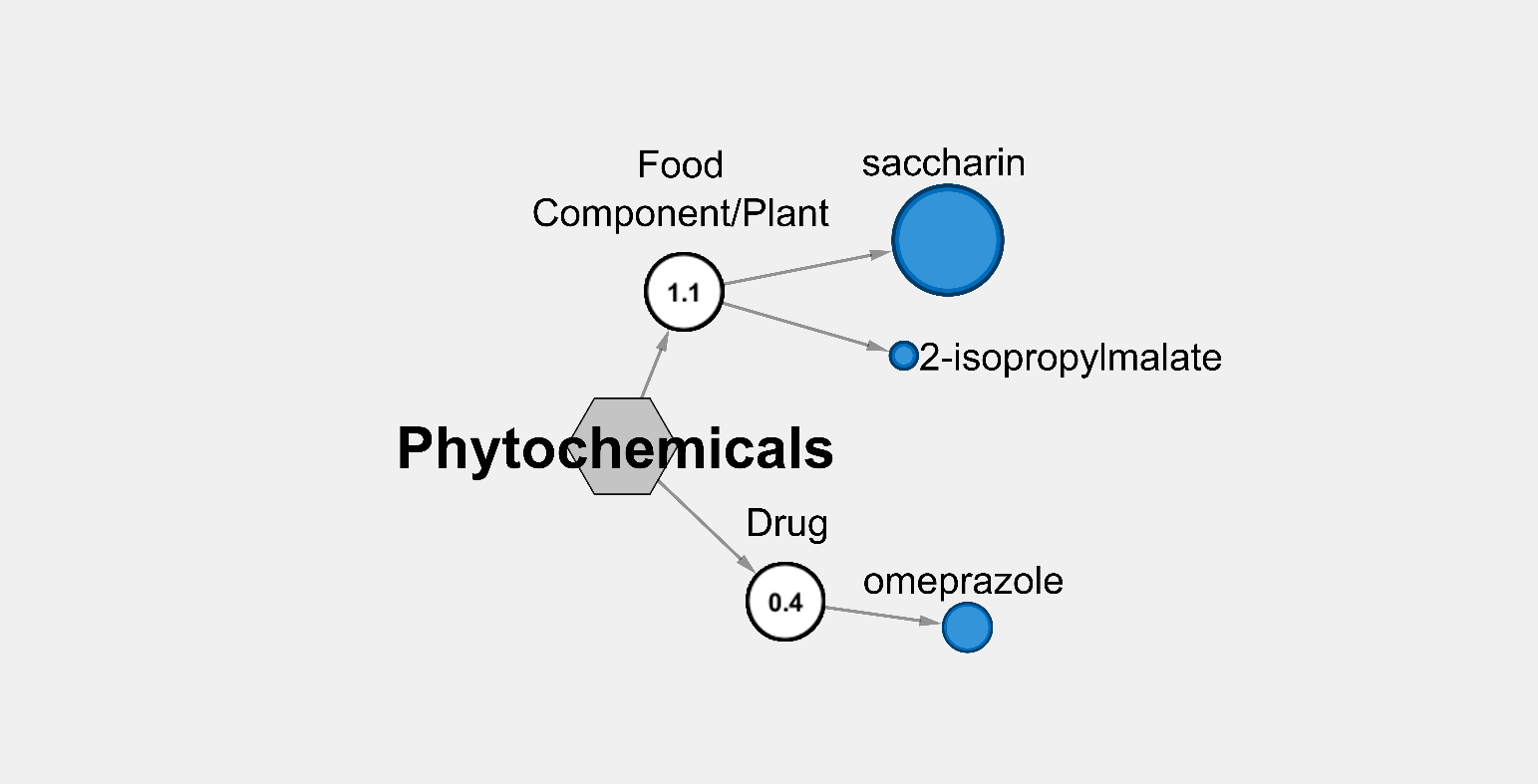
**

**(d)**

**
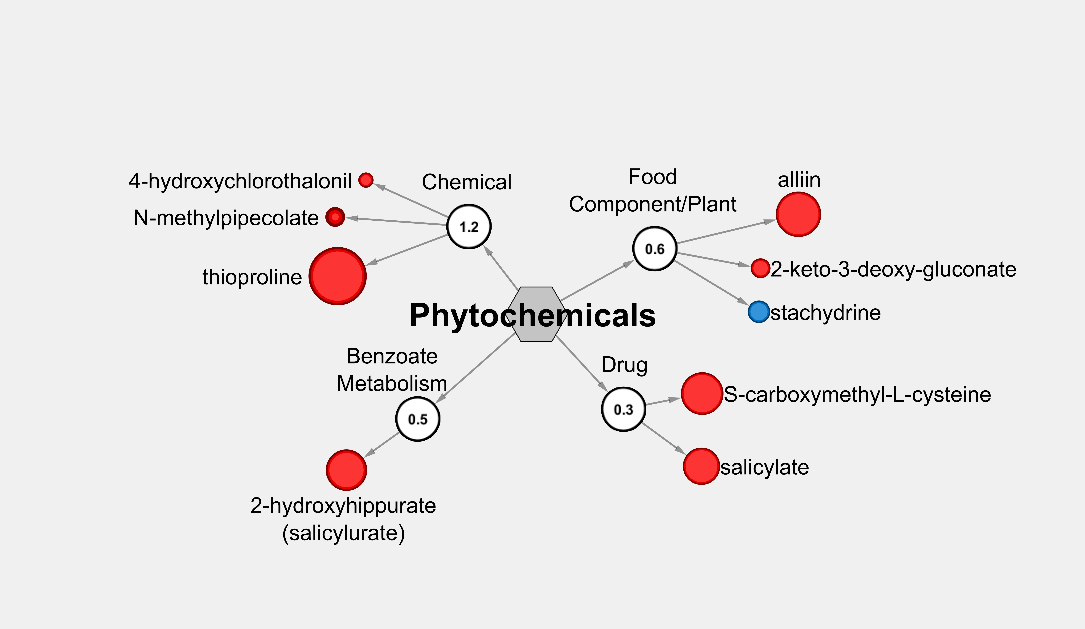
**

**(e)**

**
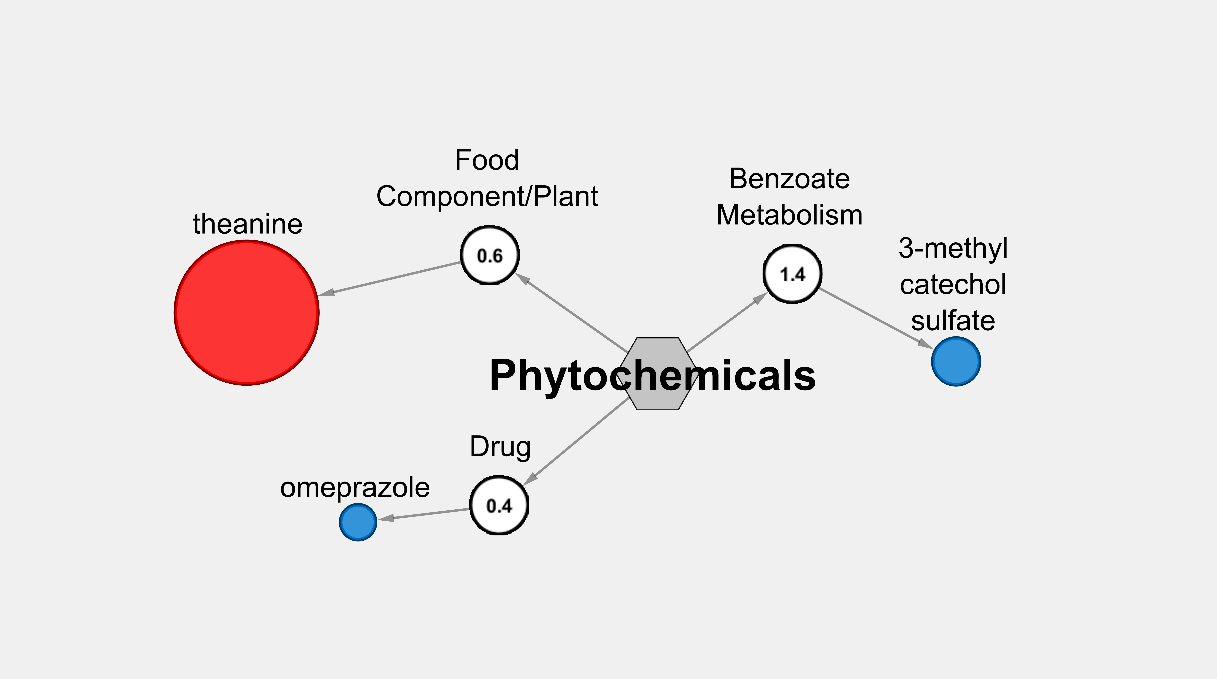
**

**(f)**

**
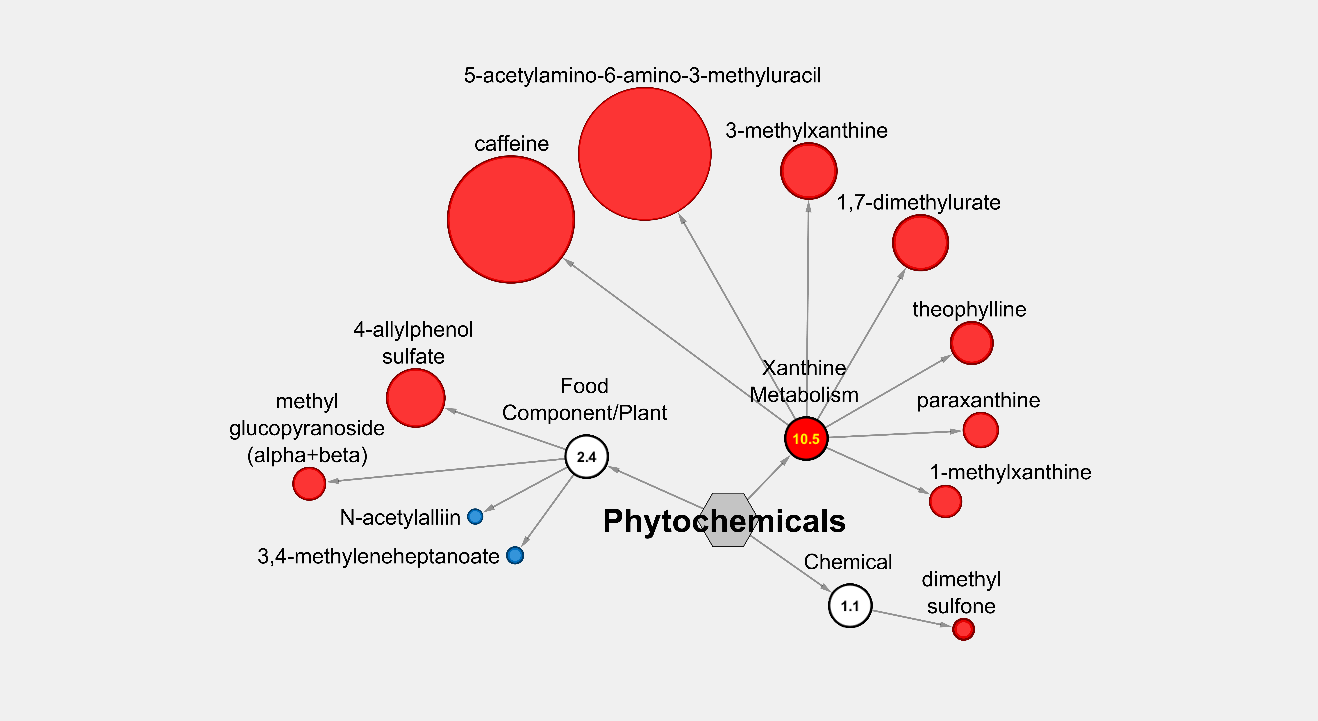
**

## Supplementary Tables

| **Supplemental Table 1 Other Modulated Plasma Metabolites Following Navy Bean and/or**  **Rice Bran Consumption for 4 Weeks Compared to Baseline^a^** | | | | | | |
| --- | --- | --- | --- | --- | --- | --- |
| **Metabolic Pathway** | | **Metabolite** | **Fold-Change *vs*. Baseline^b^** | | | |
|  |  |  | **Control** | **Navy Bean** | **Rice Bran** | **Navy Bean + Rice Bran** |
| **Amino Acid** | | | | | | |
|  | Gly, Ser & Thr Metabolism | glycine | 1.08 | 1.03 | **1.18↑*** | 1.03 |
|  |  | N-acetylglycine | **0.77↓*** | **0.76↓**** | 1.18 | 0.93 |
|  |  | dimethylglycine | 1.29 | 0.93 | **1.65↑*** | 0.93 |
|  |  | serine | 1.13 | 0.94 | **1.79↑**** | 0.89 |
|  | Ala & Asp Metabolism | N-acetylasparagine | 1.03 | 0.87 | **1.31↑*** | 1.10 |
|  |  | N-acetylaspartate | 1.05 | 1.02 | **0.89↓*** | 1.00 |
|  | Glu Metabolism | glutamine | 1.02 | 1.03 | **1.12↑**** | 0.99 |
|  | His Metabolism | histidine | 1.04 | 0.99 | **1.14↑*** | 1.03 |
|  |  | N-acetyl-3-methylhistidine | 1.11 | **1.79↑*** | 0.86 | 1.00 |
|  | Lys Metabolism | lysine | 1.11 | 1.00 | **1.12↑*** | 1.04 |
|  |  | pipecolate | 1.85 | **2.86↑*** | 1.52 | 1.53 |
|  | Phe & Tyr Metabolism | phenylalanine | 1.07 | 0.97 | **1.11↑*** | 1.04 |
|  |  | tyrosine | **1.18↑*** | 0.94 | 1.13 | 1.08 |
|  |  | N-acetyltyrosine | 1.20 | 0.99 | **1.50↑*** | 1.11 |
|  |  | 4-hydroxyphenylpyruvate | 1.14 | 1.05 | **1.63↑*** | 0.96 |
|  |  | 3-(4-hydroxyphenyl) lactate | 1.00 | 0.98 | 1.05 | **1.17↑*** |
|  |  | homovanillate | 1.49 | 1.19 | **2.32↑**** | 1.02 |
|  |  | N-formylphenylalanine | **1.15↑*** | 1.96 | 1.16 | 1.14 |
|  |  | 5-bromotryptophan | 1.08 | 1.06 | **1.15↑*** | 1.01 |
|  | Trp Metabolism | indolepropionate | **1.91↑*** | 1.60 | 1.11 | 1.12 |
|  |  | kynurenate | 1.04 | **0.87↓*** | 1.15 | 1.01 |
|  |  | 5-hydroxyindoleacetate | **1.47↑*** | 0.97 | 0.91 | 1.08 |
|  |  | thioproline | 1.61 | 1.03 | **4.29↑**** | 0.73 |
|  | Leu, Ile & Val Metabolism | leucine | 1.07 | 0.94 | **1.14↑*** | 1.01 |
|  |  | isovalerylglycine | **3.83↑**** | 1.57 | 1.25 | 0.86 |
|  |  | 2,3-dihydroxy-2-methylbutyrate | 1.27 | **1.57↑*** | 0.83 | 1.09 |
|  |  | 2-hydroxy-3-methylvalerate | 0.98 | **1.32↑*** | 0.95 | 1.00 |
|  |  | N-acetylvaline | 1.41 | 0.97 | 1.32 | **0.76↓*** |
|  |  | methylsuccinoylcarnitine (1) | 1.09 | **0.85↓*** | 1.10 | 1.06 |
|  | Met, Cys, SAM and Taurine Metabolism | methionine sulfone | 1.27 | 0.95 | **3.34↑**** | 0.85 |
|  |  | methionine sulfoxide | 1.27 | 0.88 | **1.48↑*** | 0.98 |
|  |  | cystathionine | **2.85↑**** | 1.56 | 1.81 | 1.35 |
|  |  | cysteine | 1.02 | 0.97 | **1.20↑*** | 1.03 |
|  |  | S-methylcysteine | 1.31 | **2.12↑**** | 1.31 | 1.33 |
|  |  | S-methylcysteine sulfoxide | 1.34 | **2.19↑**** | 1.62 | 1.79 |
|  |  | cysteine sulfinic acid | **0.76↓*** | 0.87 | 1.00 | 1.21 |
|  | Urea cycle; Arg & Pro Metabolism | arginine | 0.99 | 1.01 | **0.92↓*** | 1.07 |
|  |  | proline | 1.02 | 0.98 | **1.20↑**** | 1.07 |
|  |  | citrulline | 1.09 | 1.04 | **1.23↑*** | 0.94 |
|  |  | dimethylarginine (SDMA + ADMA) | 1.01 | 0.98 | **1.09↑*** | 1.01 |
|  |  | N-delta-acetylornithine | 1.01 | **1.33↑*** | 0.94 | 1.10 |
|  |  | N-alpha-acetylornithine | 1.21 | **0.81↓*** | 1.04 | 1.21 |
|  |  | N-acetylcitrulline | 1.34 | **0.80↓*** | 1.02 | 1.65 |
|  | Creatine Metabolism | creatinine | 0.97 | 0.98 | **1.06↑**** | 1.00 |
|  |  | guanidinoacetate | 1.19 | 1.09 | **1.58↑**** | 1.04 |
|  | Polyamine Metabolism | spermidine | 3.20 | 2.59 | **0.73↓*** | 2.96 |
|  | Glutathione Metabolism | cysteinylglycine | 1.03 | 1.13 | **1.64↑**** | 1.19 |
|  |  | 5-oxoproline | 1.04 | 1.00 | **1.23↑**** | 1.01 |
| **Peptide** | | | | | | |
|  | Gamma-glutamyl Amino Acid | gamma-glutamylglutamate | 1.16 | 0.93 | **2.17↑***** | 0.86 |
|  |  | gamma-glutamylglutamine | 1.02 | 1.00 | **1.45↑***** | 0.98 |
|  |  | gamma-glutamylhistidine | 1.20 | 0.94 | **1.53↑*** | 1.16 |
|  |  | gamma-glutamylleucine | 1.05 | 1.04 | **1.36↑**** | 0.96 |
|  |  | gamma-glutamyl-alpha-lysine | 1.09 | 0.98 | **1.21↑*** | 1.07 |
|  |  | gamma-glutamyl-epsilon-lysine | **0.84↓*** | 1.00 | 1.09 | 1.34 |
|  |  | gamma-glutamylphenylalanine | 1.06 | 1.02 | **1.68↑**** | 1.44 |
|  |  | gamma-glutamylthreonine | 1.46 | 1.05 | **2.27↑*** | 0.91 |
|  |  | gamma-glutamyltryptophan | 1.02 | 0.95 | **1.32↑*** | 0.90 |
|  |  | gamma-glutamyltyrosine | 1.12 | 0.93 | 1.16 | **1.23↑*** |
|  |  | gamma-glutamylvaline | 1.04 | 1.02 | **1.28↑*** | 1.02 |
|  | Fibrinogen Cleavage Peptide | DSGEGDFXAEGGGVR | 3.04 | 1.00 | 1.36 | **5.15↑*** |
|  | Acetylated Peptides | phenylacetylglutamate | 1.00 | 1.33 | 0.91 | **1.87↑*** |
|  |  | phenylacetylglutamine | **0.76↓*** | 0.91 | 0.97 | 1.72 |
| **Carbohydrate** | | | | | | |
|  | Glycolysis, Gluconeogenesis, and Pyruvate Metabolism | glucose | 0.96 | 1.05 | **0.91↓*** | 1.08 |
|  |  | glycerate | 0.89 | 0.94 | **0.80↓**** | 1.05 |
|  | Pentose Metabolism | arabitol/xylitol | 0.99 | 1.00 | **1.17↑**** | 1.02 |
|  |  | arabonate/xylonate | 0.86 | 1.08 | **0.84↓*** | 1.13 |
|  | Disaccharides and Oligosaccharides | sucrose | 0.77 | 1.89 | 0.80 | **2.60↑*** |
|  | Aminosugar Metabolism | erythronate | 0.99 | 1.01 | **0.90↓*** | 0.99 |
|  |  | N-acetylglucosamine/N-acetylgalactosamine | 1.04 | 1.00 | **1.18↑**** | 0.99 |
| **Energy** | | | | | | |
|  | TCA Cycle | citrate | 1.01 | 0.97 | **1.09↑**** | 0.97 |
|  |  | fumarate | 2.65 | 2.37 | **0.41↓**** | 1.41 |
| **Nucleotide** | | | | | | |
|  | Purine Metabolism, (Hypo)Xanthine/Inosine containing | hypoxanthine | **2.15↑*** | 1.04 | **3.15↑*** | 0.90 |
|  |  | N1-methylinosine | 1.05 | 0.90 | **1.36↑**** | 0.97 |
|  |  | allantoin | **0.82↓**** | **0.76↓***** | **0.71↓***** | 1.07 |
|  | Purine Metabolism, Adenine containing | adenosine 3',5'-cyclic monophosphate | **0.82↓*** | 1.19 | 1.36 | 1.10 |
|  |  | N6-carbamoylthreonyladenosine | 1.05 | 1.04 | **1.23↑*** | 1.05 |
|  | Purine Metabolism, Guanine containing | guanosine | **15.48↑*** | 0.93 | 2.95 | 1.06 |
|  | Pyrimidine Metabolism, Orotate containing | dihydroorotate | 1.40 | 2.25 | **2.58↑*** | 0.93 |
|  | Pyrimidine Metabolism, Uracil containing | uridine | 1.14 | 0.96 | **1.27↑*** | 1.00 |
|  |  | N-acetyl-beta-alanine | 1.06 | 0.93 | **1.18↑*** | 1.01 |
|  | Pyrimidine Metabolism, Cytidine containing | cytidine | 3.46 | 3.67 | **0.65↓*** | 3.02 |
| **Cofactors/Vitamins** | | | | | | |
|  | Nicotinate and Nicotinamide Metabolism | trigonelline (N'-methylnicotinate) | 2.42 | **7.65↑**** | 2.25 | 1.15 |
|  | Pantothenate and CoA Metabolism | pantothenate | 1.05 | 0.92 | **1.32↑**** | 1.00 |
|  | Ascorbate and Aldarate Metabolism | ascorbate (Vitamin C) | 1.25 | 1.43 | 3.55 | **1.99↑*** |
|  | Tocopherol Metabolism | alpha-CEHC sulfate | 0.90 | 1.60 | 2.11 | **0.70↓*** |
|  | Hemoglobin and Porphyrin Metabolism | bilirubin (Z,Z) | 1.46 | 1.34 | **2.20↑**** | 0.93 |
|  |  | bilirubin (E,E) | **0.69↓**** | **0.64↓***** | 1.06 | 1.03 |
|  |  | bilirubin (E,Z or Z,E) | 1.19 | 1.20 | **1.70↑**** | 0.93 |
|  | Vitamin B6 Metabolism | pyridoxal | 1.32 | 1.67 | **3.96↑*** | 1.96 |
| **Phytochemicals/Other** | | | | | | |
|  | Benzoate Metabolism | 2-hydroxyhippurate (salicylurate) | 1.88 | 1.34 | **3.05↑*** | 3.82 |
|  | Xanthine Metabolism | caffeine | 5.68 | **0.50↓*** | 3.94 | **9.59↑*** |
|  |  | paraxanthine | **2.65↑*** | **0.65↓*** | 1.61 | **2.73↑*** |
|  |  | theophylline | 2.60 | **0.68↓*** | 1.33 | **3.30↑*** |
|  |  | 1,3-dimethylurate | 1.25 | **0.79↓*** | 1.22 | 1.29 |
|  |  | 1,7-dimethylurate | 2.96 | **0.71↓*** | 1.70 | **4.29↑**** |
|  |  | 1-methylxanthine | 1.17 | 0.97 | 1.08 | **2.50↑*** |
|  |  | 3-methylxanthine | 2.24 | **0.64↓*** | 2.03 | **4.32↑*** |
|  |  | 5-acetylamino-6-amino-3-methyluracil | **7.28↑*** | **0.60↓*** | 3.06 | **10.85↑*** |
|  | Tobacco Metabolite | cotinine | **0.87↓*** | 1.00 | 1.00 | 1.00 |
|  | Food Component/Plant | retinal | **0.73↓*** | 1.21 | 1.04 | 1.65 |
|  |  | alliin | 1.88 | 1.17 | **3.34↑*** | 1.12 |
|  |  | N-acetylalliin | **1.98↑*** | 1.09 | 1.20 | **0.82↓*** |
|  |  | ferulic acid 4-sulfate | 1.11 | **4.62↑*** | 1.23 | 0.91 |
|  |  | stachydrine | 1.44 | 3.46 | **0.60↓*** | 2.08 |
|  |  | 4-allylphenol sulfate | **9.19↑*** | 2.54 | 4.66 | **4.47↑*** |
|  |  | methyl glucopyranoside (alpha + beta) | 1.55 | 1.67 | 1.00 | **2.55↑*** |
|  |  | 4-vinylguaiacol sulfate | 1.12 | **20.17↑*** | 1.64 | 2.40 |
|  |  | umbelliferone sulfate | **6.74↑*** | 0.95 | 1.00 | 0.86 |
|  |  | 2-keto-3-deoxy-gluconate | 1.02 | 1.02 | **1.46↑*** | 1.32 |
|  |  | 3,4-methyleneheptanoate | 0.82 | 1.13 | 1.24 | **0.73↓**** |
|  | Drug/Plant | omeprazole | **2.14↑*** | 1.00 | 1.00 | 1.00 |
|  |  | salicylate | 1.22 | 1.25 | **2.74↑*** | 3.03 |
|  |  | S-carboxymethyl-L-cysteine | 1.78 | 1.00 | **3.14↑*** | 1.13 |
|  | Chemical | dimethyl sulfone | 1.20 | **1.54↑*** | 1.28 | **1.72↑**** |
|  |  | ectoine | 1.60 | **23.49↑*** | 3.15 | 2.30 |
|  |  | N-methylpipecolate | **0.79↓**** | 1.02 | **1.46↑***** | 0.96 |
|  |  | 4-hydroxychlorothalonil | 1.00 | **0.92↓*** | **1.12↑*** | 0.95 |

Alpha-CEHC, 2, 5, 7, 8-tetramethyl-2-(2'-carboxyethyl)-6-hydroxychroman; ADMA, asymmetric dimethylarginine; Ala, alanine; Arg, arginine; Asp, aspartate; Cys, cysteine; Glu, glutamate; Gly, glycine; His, histidine; Ile, isoleucine; Leu, leucine; Lys, lysine; Met, methionine; Phe, phenylalanine; Pro, proline; SAM, S-adenosyl methionine; SDMA, symmetric dimethylarginine; Ser, serine; TCA, tricarboxylic acid; Thr, threonine; Trp, tryptophan; Tyr, tyrosine; Val, valine.

^a^Metabolites that were detected in plasma but non-significant (p>0.05) in dietary groups compared to respective baseline are not presented in this table. ^b^Values presented are mean metabolite ratios/fold-changes. Statistically-significantly increased (↑) fold-changes are bolded and highlighted in red, and statistically-significantly decreased (↓) fold-changes are bolded and highlighted in blue (^*^p≤0.05; ^**^p≤0.01; ^***^p≤0.001).

| **Supplemental Table 2 Relative Abundance of Food Metabolites With Dual Plasma Detection from Children Consuming**  **Navy Bean and/or Rice Bran for 4 Weeks** | | | | |
| --- | --- | --- | --- | --- |
| **Metabolic Pathway** | **Metabolite** | **HMDB** | **Diet Intervention** | |
|  |  |  | **Navy Bean** | **Rice Bran** |
| **Amino Acid** | | | | |
| Ala & Asp Metabolism | alanine | [HMDB00161](http://www.hmdb.ca/metabolites/HMDB00161) | 22,623,284 | 154,873,584 |
|  | asparagine | [HMDB00168](http://www.hmdb.ca/metabolites/HMDB00168) | 312,901,248 | 854,978,432 |
|  | aspartate | [HMDB00191](http://www.hmdb.ca/metabolites/HMDB00191) | 178,237,616 | 313,753,824 |
|  | N-acetylalanine | [HMDB00766](http://www.hmdb.ca/metabolites/HMDB00766) | 256,331 | - |
|  | N-acetylasparagine | [HMDB06028](http://www.hmdb.ca/metabolites/HMDB06028) | 710,549 | 2,219,634 |
|  | N-acetylaspartate | [HMDB00812](http://www.hmdb.ca/metabolites/HMDB00812) | 411,785 | - |
| Creatine Metabolism | creatine | [HMDB00064](http://www.hmdb.ca/metabolites/HMDB00064) | - | 691,673 |
|  | guanidinoacetate | [HMDB00128](http://www.hmdb.ca/metabolites/HMDB00128) | - | 1,080,912 |
| Glu Metabolism | glutamate | [HMDB00148](http://www.hmdb.ca/metabolites/HMDB00148) | 332,101,376 | 949,940,800 |
|  | glutamine | [HMDB00641](http://www.hmdb.ca/metabolites/HMDB00641) | 8,096,717 | 176,320,032 |
|  | N-acetylglutamate | [HMDB01138](http://www.hmdb.ca/metabolites/HMDB01138) | 444,456 | 2,615,805 |
|  | N-acetylglutamine | [HMDB06029](http://www.hmdb.ca/metabolites/HMDB06029) | - | 746,564 |
|  | pyroglutamine |  | 3,915,682 | 21,470,568 |
| Glutathione Metabolism | 5-oxoproline | [HMDB00267](http://www.hmdb.ca/metabolites/HMDB00267) | 247,470,496 | 13,207,618 |
|  | cysteine-glutathione disulfide | [HMDB00656](http://www.hmdb.ca/metabolites/HMDB00656) | - | 1,270,450 |
|  | glutathione, oxidized | [HMDB03337](http://www.hmdb.ca/metabolites/HMDB03337) | - | 73,523,688 |
|  | ophthalmate | [HMDB05765](http://www.hmdb.ca/metabolites/HMDB05765) | - | 2,325,022 |
| Gly, Ser & Thr Metabolism | betaine | [HMDB00043](http://www.hmdb.ca/metabolites/HMDB00043) | 10,722,703 | 304,460,288 |
|  | glycine | [HMDB00123](http://www.hmdb.ca/metabolites/HMDB00123) | 2,313,037 | 2,299,147 |
|  | N-acetylglycine | [HMDB00532](http://www.hmdb.ca/metabolites/HMDB00532) | - | 439,641 |
|  | N-acetylserine | [HMDB02931](http://www.hmdb.ca/metabolites/HMDB02931) | 338,999 | 156,770 |
|  | O-acetylhomoserine |  | 91,969 | 127,549 |
|  | serine | [HMDB00187](http://www.hmdb.ca/metabolites/HMDB00187) | 5,971,022 | 85,386,120 |
|  | threonine | [HMDB00167](http://www.hmdb.ca/metabolites/HMDB00167) | 39,759,256 | 43,382,872 |
| Guanidino & Acetamido Metabolism | 4-guanidinobutanoate | [HMDB03464](http://www.hmdb.ca/metabolites/HMDB03464) | 11,928,345 | 122,057,408 |
| His Metabolism | 3-methylhistidine | [HMDB00479](http://www.hmdb.ca/metabolites/HMDB00479) | - | 67,425 |
|  | 4-imidazoleacetate | [HMDB02024](http://www.hmdb.ca/metabolites/HMDB02024) | - | 3,308,998 |
|  | histidine | [HMDB00177](http://www.hmdb.ca/metabolites/HMDB00177) | 16,185,785 | 8,606,607 |
|  | imidazole lactate | [HMDB02320](http://www.hmdb.ca/metabolites/HMDB02320) | - | 432,494 |
|  | imidazole propionate | [HMDB02271](http://www.hmdb.ca/metabolites/HMDB02271) | 202,127 | - |
|  | N-acetylhistidine | [HMDB32055](http://www.hmdb.ca/metabolites/HMDB32055) | - | 10,576,467 |
|  | trans-urocanate | [HMDB00301](http://www.hmdb.ca/metabolites/HMDB00301) | - | 709,868 |
| Leu, Ile & Val Metabolism | 2-hydroxy-3-methylvalerate | [HMDB00317](http://www.hmdb.ca/metabolites/HMDB00317) | 319,706 | 337,822 |
|  | 3-hydroxy-2-ethylpropionate | [HMDB00396](http://www.hmdb.ca/metabolites/HMDB00396) | - | 144,142 |
|  | 3-methyl-2-oxobutyrate | [HMDB00019](http://www.hmdb.ca/metabolites/HMDB00019) | 305,952 | - |
|  | 3-methyl-2-oxovalerate | [HMDB03736](http://www.hmdb.ca/metabolites/HMDB03736) | 395,053 | 278,537 |
|  | 4-methyl-2-oxopentanoate | [HMDB00695](http://www.hmdb.ca/metabolites/HMDB00695) | 457,715 | 156,834 |
|  | alpha-hydroxyisocaproate | [HMDB00746](http://www.hmdb.ca/metabolites/HMDB00746) | 469,919 | 195,324 |
|  | alpha-hydroxyisovalerate | [HMDB00407](http://www.hmdb.ca/metabolites/HMDB00407) | - | 74,789 |
|  | beta-hydroxyisovalerate | [HMDB00754](http://www.hmdb.ca/metabolites/HMDB00754) | 34,909 | 520,932 |
|  | ethylmalonate | [HMDB00622](http://www.hmdb.ca/metabolites/HMDB00622) | - | 3,100,378 |
|  | isobutyrylcarnitine | [HMDB00736](http://www.hmdb.ca/metabolites/HMDB00736) | - | 324,838 |
|  | isobutyrylglycine | [HMDB00730](http://www.hmdb.ca/metabolites/HMDB00730) | 143,829 | 220,797 |
|  | isoleucine | [HMDB00172](http://www.hmdb.ca/metabolites/HMDB00172) | 73,242,192 | 122,538,464 |
|  | isovalerate | [HMDB00718](http://www.hmdb.ca/metabolites/HMDB00718) | 910,678 | 1,178,115 |
|  | isovalerylcarnitine | [HMDB00688](http://www.hmdb.ca/metabolites/HMDB00688) | - | 295,877 |
|  | isovalerylglycine | [HMDB00678](http://www.hmdb.ca/metabolites/HMDB00678) | 88,008 | 258,226 |
|  | leucine | [HMDB00687](http://www.hmdb.ca/metabolites/HMDB00687) | 154,497,152 | 133,660,944 |
|  | methylsuccinate | [HMDB01844](http://www.hmdb.ca/metabolites/HMDB01844) | 1,049,436 | 5,893,828 |
|  | N-acetylisoleucine |  | - | 132,477 |
|  | N-acetylleucine | [HMDB11756](http://www.hmdb.ca/metabolites/HMDB11756) | - | 151,219 |
|  | N-acetylvaline | [HMDB11757](http://www.hmdb.ca/metabolites/HMDB11757) | 137,856 | 221,255 |
|  | valine | [HMDB00883](http://www.hmdb.ca/metabolites/HMDB00883) | 100,709,928 | 135,666,352 |
| Lys Metabolism | 2-aminoadipate | [HMDB00510](http://www.hmdb.ca/metabolites/HMDB00510) | 631,719 | 11,574,106 |
|  | glutarate (pentanedioate) | [HMDB00661](http://www.hmdb.ca/metabolites/HMDB00661) | 138,430 | 551,722 |
|  | lysine | [HMDB00182](http://www.hmdb.ca/metabolites/HMDB00182) | 13,117,043 | 23,819,276 |
|  | N2-acetyllysine | [HMDB00446](http://www.hmdb.ca/metabolites/HMDB00446) | 105,633 | 579,540 |
|  | N6-acetyllysine | [HMDB00206](http://www.hmdb.ca/metabolites/HMDB00206) | 1,653,600 | 1,393,616 |
|  | N-6-trimethyllysine | [HMDB01325](http://www.hmdb.ca/metabolites/HMDB01325) | 17,655,794 | 42,744,088 |
|  | pipecolate | [HMDB00070](http://www.hmdb.ca/metabolites/HMDB00070) | 8,570,766,336 | 20,772,442 |
| Met, Cys, SAM & Taurine Metabolism | 2-hydroxybutyrate | [HMDB00008](http://www.hmdb.ca/metabolites/HMDB00008) | 114,593 | 155,680 |
|  | cysteine | [HMDB00574](http://www.hmdb.ca/metabolites/HMDB00574) | 45,081 | 65,957 |
|  | methionine | [HMDB00696](http://www.hmdb.ca/metabolites/HMDB00696) | 17,036,198 | 43,163,732 |
|  | methionine sulfone |  | 445,226 | 103,816 |
|  | methionine sulfoxide | [HMDB02005](http://www.hmdb.ca/metabolites/HMDB02005) | 213,844 | 15,724,799 |
|  | N-acetylmethionine | [HMDB11745](http://www.hmdb.ca/metabolites/HMDB11745) | 553,640 | 444,416 |
|  | N-acetyltaurine |  | - | 437,749 |
|  | S-adenosylhomocysteine | [HMDB00939](http://www.hmdb.ca/metabolites/HMDB00939) | 2,650,590 | 2,438,966 |
|  | S-methylcysteine | [HMDB02108](http://www.hmdb.ca/metabolites/HMDB02108) | 226,654,064 | - |
|  | taurine | [HMDB00251](http://www.hmdb.ca/metabolites/HMDB00251) | - | 1,720,630 |
| Phe & Tyr Metabolism | 3-(4-hydroxyphenyl)lactate | [HMDB00755](http://www.hmdb.ca/metabolites/HMDB00755) | 174,583 | 422,103 |
|  | 3-(4-hydroxyphenyl)propionate | [HMDB02199](http://www.hmdb.ca/metabolites/HMDB02199) | - | 91,241 |
|  | 3-methoxytyrosine | [HMDB01434](http://www.hmdb.ca/metabolites/HMDB01434) | 1,171,878 | 95,780 |
|  | 3-phenylpropionate (hydrocinnamate) | [HMDB00764](http://www.hmdb.ca/metabolites/HMDB00764) | - | 79,594 |
|  | 4-hydroxyphenylpyruvate | [HMDB00707](http://www.hmdb.ca/metabolites/HMDB00707) | 266,042 | 76,873 |
|  | N-acetylphenylalanine | [HMDB00512](http://www.hmdb.ca/metabolites/HMDB00512) | 327,515 | 248,397 |
|  | N-acetyltyrosine | [HMDB00866](http://www.hmdb.ca/metabolites/HMDB00866) | - | 124,359 |
|  | N-formylphenylalanine |  | 276,093 | 295,357 |
|  | phenylalanine | [HMDB00159](http://www.hmdb.ca/metabolites/HMDB00159) | 249,640,912 | 146,600,208 |
|  | phenyllactate | [HMDB00779](http://www.hmdb.ca/metabolites/HMDB00779) | 169,872 | 340,857 |
|  | phenylpyruvate | [HMDB00205](http://www.hmdb.ca/metabolites/HMDB00205) | 195,117 | - |
|  | tyrosine | [HMDB00158](http://www.hmdb.ca/metabolites/HMDB00158) | 27,884,908 | 106,920,152 |
| Polyamine Metabolism | 4-acetamidobutanoate | [HMDB03681](http://www.hmdb.ca/metabolites/HMDB03681) | 1,846,783 | 2,554,135 |
|  | 5-methylthioadenosine | [HMDB01173](http://www.hmdb.ca/metabolites/HMDB01173) | 3,644,063 | - |
|  | N-acetylputrescine | [HMDB02064](http://www.hmdb.ca/metabolites/HMDB02064) | 207,892 | 1,170,238 |
|  | spermidine | [HMDB01257](http://www.hmdb.ca/metabolites/HMDB01257) | 21,052,426 | 4,807,387 |
| Trp Metabolism | indole-3-carboxylic acid | [HMDB03320](http://www.hmdb.ca/metabolites/HMDB03320) | 118,743 | 622,346 |
|  | indoleacetate | [HMDB00197](http://www.hmdb.ca/metabolites/HMDB00197) | - | 2,258,563 |
|  | kynurenate | [HMDB00715](http://www.hmdb.ca/metabolites/HMDB00715) | - | 322,359 |
|  | kynurenine | [HMDB00684](http://www.hmdb.ca/metabolites/HMDB00684) | 333,660 | 341,555 |
|  | N-acetyltryptophan | [HMDB13713](http://www.hmdb.ca/metabolites/HMDB13713) | 228,472 | - |
|  | picolinate | [HMDB02243](http://www.hmdb.ca/metabolites/HMDB02243) | - | 265,771 |
|  | serotonin | [HMDB00259](http://www.hmdb.ca/metabolites/HMDB00259) | - | 5,753,170 |
|  | tryptophan | [HMDB00929](http://www.hmdb.ca/metabolites/HMDB00929) | 207,239,376 | 233,788,992 |
|  | xanthurenate | [HMDB00881](http://www.hmdb.ca/metabolites/HMDB00881) | - | 328,114 |
| Urea cycle; Arg & Pro Metabolism | arginine | [HMDB00517](http://www.hmdb.ca/metabolites/HMDB00517) | 483,593,984 | 157,627,392 |
|  | citrulline | [HMDB00904](http://www.hmdb.ca/metabolites/HMDB00904) | 8,234,870 | 3,331,102 |
|  | dimethylarginine (SDMA + ADMA) | [HMDB01539](http://www.hmdb.ca/metabolites/HMDB01539) | 618,972 | 17,090,950 |
|  | homocitrulline | [HMDB00679](http://www.hmdb.ca/metabolites/HMDB00679) | 3,120,438 | 1,874,632 |
|  | N-acetylarginine | [HMDB04620](http://www.hmdb.ca/metabolites/HMDB04620) | 956,820 | 1,132,691 |
|  | N-delta-acetylornithine |  | 131,186,960 | 3,704,738 |
|  | N-methylproline |  | 3,593,033 | 6,995,909 |
|  | ornithine | [HMDB03374](http://www.hmdb.ca/metabolites/HMDB03374) | 1,117,834 | 1,732,765 |
|  | proline | [HMDB00162](http://www.hmdb.ca/metabolites/HMDB00162) | 79,332,312 | 176,096,400 |
|  | trans-4-hydroxyproline | [HMDB00725](http://www.hmdb.ca/metabolites/HMDB00725) | 3,733,790 | 5,215,292 |
| **Carbohydrate** | | | | |
| Advanced Glycation End-product | N6-carboxymethyllysine |  | - | 942,803 |
| Aminosugar Metabolism | erythronate | [HMDB00613](http://www.hmdb.ca/metabolites/HMDB00613) | 7,839,455 | 21,380,400 |
|  | glucuronate | [HMDB00127](http://www.hmdb.ca/metabolites/HMDB00127) | - | 858,124 |
|  | N-acetylgalactosamine | [HMDB00212](http://www.hmdb.ca/metabolites/HMDB00212) | 302,439 | 61,414 |
|  | N-acetylglucosamine | [HMDB00215](http://www.hmdb.ca/metabolites/HMDB00215) | 72,471 | 89,155 |
| Disaccharides & Oligosaccharides | sucrose | [HMDB00258](http://www.hmdb.ca/metabolites/HMDB00258) | 21,856,802 | 80,941,968 |
| Fructose, Mannose & Galactose Metabolism | fructose | [HMDB00660](http://www.hmdb.ca/metabolites/HMDB00660) | 10,205,347 | 19,963,404 |
|  | mannitol | [HMDB00765](http://www.hmdb.ca/metabolites/HMDB00765) | 98,908 | 440,706 |
|  | mannose | [HMDB00169](http://www.hmdb.ca/metabolites/HMDB00169) | 533,469 | - |
|  | sorbitol | [HMDB00247](http://www.hmdb.ca/metabolites/HMDB00247) | 199,290 | 1,976,820 |
| Glycolysis, Gluconeogenesis, & Pyruvate Metabolism | glucose | [HMDB00122](http://www.hmdb.ca/metabolites/HMDB00122) | 6,482,590 | 39,644,908 |
|  | glycerate | [HMDB00139](http://www.hmdb.ca/metabolites/HMDB00139) | 6,059,944 | 15,721,188 |
|  | lactate | [HMDB00190](http://www.hmdb.ca/metabolites/HMDB00190) | 5,391,952 | 1,094,495 |
|  | pyruvate | [HMDB00243](http://www.hmdb.ca/metabolites/HMDB00243) | 2,237,065 | 2,489,029 |
| Pentose Metabolism | arabinose | [HMDB00646](http://www.hmdb.ca/metabolites/HMDB00646) | 241,577 | 274,619 |
|  | arabitol | [HMDB00568](http://www.hmdb.ca/metabolites/HMDB00568) | - | 602,780 |
|  | ribitol | [HMDB00508](http://www.hmdb.ca/metabolites/HMDB00508) | 375,254 | 1,082,531 |
|  | ribonate | [HMDB00867](http://www.hmdb.ca/metabolites/HMDB00867) | 3,172,713 | 15,624,751 |
|  | xylitol | [HMDB02917](http://www.hmdb.ca/metabolites/HMDB02917) | 35,227 | 84,737 |
|  | xylonate | [HMDB60256](http://www.hmdb.ca/metabolites/HMDB60256) | 95,690 | 236,192 |
|  | xylose | [HMDB00098](http://www.hmdb.ca/metabolites/HMDB00098) | 1,208,074 | 188,364 |
| **Cofactors and Vitamins** | | | | |
| Ascorbate & Aldarate Metabolism | arabonate | [HMDB00539](http://www.hmdb.ca/metabolites/HMDB00539) | 137,147 | 336,740 |
|  | ascorbate (Vitamin C) | [HMDB00044](http://www.hmdb.ca/metabolites/HMDB00044) | - | 45,480 |
|  | gulonic acid |  | 46,846,640 | 11,174,010 |
|  | oxalate (ethanedioate) | [HMDB02329](http://www.hmdb.ca/metabolites/HMDB02329) | 9,103,215 | 94,841,352 |
|  | threonate | [HMDB00943](http://www.hmdb.ca/metabolites/HMDB00943) | 18,759,394 | 44,200,380 |
| Nicotinate & Nicotinamide Metabolism | nicotinamide | [HMDB01406](http://www.hmdb.ca/metabolites/HMDB01406) | 33,466,068 | 2,451,008 |
|  | trigonelline (N'-methylnicotinate) | [HMDB00875](http://www.hmdb.ca/metabolites/HMDB00875) | 2,776,756,224 | 743,469,632 |
| Pantothenate & CoA Metabolism | pantothenate | [HMDB00210](http://www.hmdb.ca/metabolites/HMDB00210) | 3,332,029 | 56,316,984 |
| Riboflavin Metabolism | flavin adenine dinucleotide | [HMDB01248](http://www.hmdb.ca/metabolites/HMDB01248) | - | 83,614 |
| Tocopherol Metabolism | alpha-CEHC | [HMDB01518](http://www.hmdb.ca/metabolites/HMDB01518) | 33,191 | - |
|  | alpha-tocopherol | [HMDB01893](http://www.hmdb.ca/metabolites/HMDB01893) | 10,846 | 29,343 |
|  | beta-tocopherol | [HMDB06335](http://www.hmdb.ca/metabolites/HMDB06335) | - | 5,935 |
|  | gamma-tocopherol | [HMDB01492](http://www.hmdb.ca/metabolites/HMDB01492) | 87,629 | - |
| Vitamin B6 Metabolism | pyridoxal | [HMDB01545](http://www.hmdb.ca/metabolites/HMDB01545) | 535,409 | 3,098,192 |
|  | pyridoxate | [HMDB00017](http://www.hmdb.ca/metabolites/HMDB00017) | 974,169 | 25,918,946 |
| **Energy** | | | | |
| Oxidative Phosphorylation | phosphate | [HMDB01429](http://www.hmdb.ca/metabolites/HMDB01429) | 39,321,208 | 43,862,232 |
| TCA Cycle | 2-methylcitrate | [HMDB00379](http://www.hmdb.ca/metabolites/HMDB00379) | 53,875 | 37,479 |
|  | alpha-ketoglutarate | [HMDB00208](http://www.hmdb.ca/metabolites/HMDB00208) | 4,611,359 | 44,272,720 |
|  | citrate | [HMDB00094](http://www.hmdb.ca/metabolites/HMDB00094) | 2,266,094,848 | 1,145,700,864 |
|  | fumarate | [HMDB00134](http://www.hmdb.ca/metabolites/HMDB00134) | 3,286,081 | 18,322,948 |
|  | malate | [HMDB00156](http://www.hmdb.ca/metabolites/HMDB00156) | 152,110,960 | 301,898,720 |
|  | succinate | [HMDB00254](http://www.hmdb.ca/metabolites/HMDB00254) | 22,823,878 | 149,132,480 |
| **Lipid** | | | | |
| Carnitine Metabolism | carnitine | [HMDB00062](http://www.hmdb.ca/metabolites/HMDB00062) | 5,489,914 | 8,522,175 |
|  | deoxycarnitine | [HMDB01161](http://www.hmdb.ca/metabolites/HMDB01161) | 5,917,737 | 240,476 |
| Endocannabinoid | oleic ethanolamide | [HMDB02088](http://www.hmdb.ca/metabolites/HMDB02088) | 3,581,612 | 288,953 |
|  | palmitoyl ethanolamide | [HMDB02100](http://www.hmdb.ca/metabolites/HMDB02100) | 6,014,241 | 1,690,296 |
|  | stearoyl ethanolamide | [HMDB13078](http://www.hmdb.ca/metabolites/HMDB13078) | 865,381 | - |
| Fatty Acid Metabolism (also BCAA Metabolism) | butyrylcarnitine | [HMDB02013](http://www.hmdb.ca/metabolites/HMDB02013) | - | 350,453 |
| Fatty Acid Metabolism (Acyl Carnitine) | acetylcarnitine | [HMDB00201](http://www.hmdb.ca/metabolites/HMDB00201) | 472,839 | 1,821,132 |
| Fatty Acid, Amino | 2-aminoheptanoate |  | - | 192,392 |
| Fatty Acid, Branched | 13-methylmyristic acid |  | 4,494,557 | 2,902,330 |
|  | 15-methylpalmitate (isobar with 2-methylpalmitate) |  | 4,652,166 | 4,674,955 |
|  | 17-methylstearate |  | 166,714 | 177,665 |
| Fatty Acid, Dicarboxylate | 2-hydroxyadipate | [HMDB00321](http://www.hmdb.ca/metabolites/HMDB00321) | 871,593 | 968,765 |
|  | 2-hydroxyglutarate | [HMDB00606](http://www.hmdb.ca/metabolites/HMDB00606) | 3,356,396 | 21,331,188 |
|  | 2-methylglutarate | [HMDB00752](http://www.hmdb.ca/metabolites/HMDB00752) | 311,425 | 785,973 |
|  | 3-methyladipate | [HMDB00555](http://www.hmdb.ca/metabolites/HMDB00555) | 449,103 | 853,830 |
|  | adipate | [HMDB00448](http://www.hmdb.ca/metabolites/HMDB00448) | 560,170 | 1,114,174 |
|  | azelate (nonanedioate) | [HMDB00784](http://www.hmdb.ca/metabolites/HMDB00784) | 862,324 | 12,045,892 |
|  | dodecanedioate | [HMDB00623](http://www.hmdb.ca/metabolites/HMDB00623) | 247,310 | 719,101 |
|  | hexadecanedioate | [HMDB00672](http://www.hmdb.ca/metabolites/HMDB00672) | 247,596 | 460,787 |
|  | maleate (cis-Butenedioate) | [HMDB00176](http://www.hmdb.ca/metabolites/HMDB00176) | 2,421,072 | 2,412,261 |
|  | pimelate (heptanedioate) | [HMDB00857](http://www.hmdb.ca/metabolites/HMDB00857) | - | 362,240 |
|  | sebacate (decanedioate) | [HMDB00792](http://www.hmdb.ca/metabolites/HMDB00792) | 717,683 | 981,630 |
|  | suberate (octanedioate) | [HMDB00893](http://www.hmdb.ca/metabolites/HMDB00893) | - | 1,558,489 |
|  | tetradecanedioate | [HMDB00872](http://www.hmdb.ca/metabolites/HMDB00872) | 363,911 | 406,749 |
|  | undecanedioate | [HMDB00888](http://www.hmdb.ca/metabolites/HMDB00888) | 159,669 | 1,826,683 |
| Fatty Acid, Dihydroxy | 12,13-DiHOME | [HMDB04705](http://www.hmdb.ca/metabolites/HMDB04705) | 100,619 | 123,204,584 |
|  | 9,10-DiHOME | [HMDB04704](http://www.hmdb.ca/metabolites/HMDB04704) | - | 59,692,256 |
| Fatty Acid, Monohydroxy | 13-HODE + 9-HODE |  | 6,419,312 | 7,082,311,680 |
|  | 2-hydroxydecanoate |  | 372,178 | 1,503,139 |
|  | 2-hydroxyoctanoate | [HMDB02264](http://www.hmdb.ca/metabolites/HMDB02264) | 116,929 | 216,494 |
|  | 2-hydroxypalmitate | [HMDB31057](http://www.hmdb.ca/metabolites/HMDB31057) | 1,917,395 | 2,900,843 |
|  | 2-hydroxystearate |  | 1,199,831 | 1,403,719 |
|  | 3-hydroxydecanoate | [HMDB02203](http://www.hmdb.ca/metabolites/HMDB02203) | 247,479 | 446,182 |
|  | 3-hydroxylaurate | [HMDB00387](http://www.hmdb.ca/metabolites/HMDB00387) | 479,893 | 764,962 |
|  | 3-hydroxymyristate |  | 1,755,883 | 2,469,780 |
|  | 3-hydroxyoctanoate | [HMDB01954](http://www.hmdb.ca/metabolites/HMDB01954) | 91,000 | 421,384 |
|  | 8-hydroxyoctanoate | [HMDB00711](http://www.hmdb.ca/metabolites/HMDB00711) | 141,502 | 1,151,467 |
|  | alpha-hydroxycaproate | [HMDB01624](http://www.hmdb.ca/metabolites/HMDB01624) | - | 206,678 |
| Glycerolipid Metabolism | glycerol | [HMDB00131](http://www.hmdb.ca/metabolites/HMDB00131) | - | 314,740,832 |
|  | glycerol 3-phosphate | [HMDB00126](http://www.hmdb.ca/metabolites/HMDB00126) | - | 1,958,323 |
|  | glycerophosphoglycerol |  | 1,858,490 | 1,807,928 |
| Inositol Metabolism | chiro-inositol | [HMDB34220](http://www.hmdb.ca/metabolites/HMDB34220) | - | 594,942 |
| Long Chain Fatty Acid | 10-heptadecenoate (17:1n7) | [HMDB60038](http://www.hmdb.ca/metabolites/HMDB60038) | 2,408,783 | 15,274,945 |
|  | 10-nonadecenoate (19:1n9) | [HMDB13622](http://www.hmdb.ca/metabolites/HMDB13622) | 329,098 | 3,135,657 |
|  | arachidate (20:0) | [HMDB02212](http://www.hmdb.ca/metabolites/HMDB02212) | 9,515,176 | 146,200,672 |
|  | behenate (22:0) | [HMDB00944](http://www.hmdb.ca/metabolites/HMDB00944) | 44,904 | 562,987 |
|  | eicosenoate (20:1n9 or 11) | [HMDB02231](http://www.hmdb.ca/metabolites/HMDB02231) | 3,450,340 | 153,264,032 |
|  | erucate (22:1n9) | [HMDB02068](http://www.hmdb.ca/metabolites/HMDB02068) | 676,536 | 6,825,225 |
|  | margarate (17:0) | [HMDB02259](http://www.hmdb.ca/metabolites/HMDB02259) | 15,128,211 | 24,053,634 |
|  | myristate (14:0) | [HMDB00806](http://www.hmdb.ca/metabolites/HMDB00806) | 37,736,836 | 605,839,808 |
|  | myristoleate (14:1n5) | [HMDB02000](http://www.hmdb.ca/metabolites/HMDB02000) | 1,293,227 | 71,952,344 |
|  | nonadecanoate (19:0) | [HMDB00772](http://www.hmdb.ca/metabolites/HMDB00772) | 3,270,887 | 7,285,277 |
|  | oleate (18:1n9) | [HMDB00207](http://www.hmdb.ca/metabolites/HMDB00207) | 1,810,953 | 116,389,256 |
|  | palmitate (16:0) | [HMDB00220](http://www.hmdb.ca/metabolites/HMDB00220) | 341,317,568 | 4,410,091,008 |
|  | palmitoleate (16:1n7) | [HMDB03229](http://www.hmdb.ca/metabolites/HMDB03229) | 8,392,570 | 254,569,488 |
|  | pentadecanoate (15:0) | [HMDB00826](http://www.hmdb.ca/metabolites/HMDB00826) | 12,145,118 | 26,355,804 |
|  | stearate (18:0) | [HMDB00827](http://www.hmdb.ca/metabolites/HMDB00827) | 373,335,008 | 777,727,808 |
| Lysolipid | 1-linolenoylglycerophosphocholine (18:3n3) |  | 2,115,701 | - |
|  | 1-linoleoylglycerophosphocholine (18:2n6) | [HMDB10386](http://www.hmdb.ca/metabolites/HMDB10386) | 3,258,714 | 2,582,632 |
|  | 1-linoleoylglycerophosphoethanolamine | [HMDB11507](http://www.hmdb.ca/metabolites/HMDB11507) | 4,183,353 | 1,510,417 |
|  | 1-linoleoylglycerophosphoinositol |  | 1,213,923 | 1,203,001 |
|  | 1-oleoylglycerophosphocholine (18:1) | [HMDB02815](http://www.hmdb.ca/metabolites/HMDB02815) | 2,806,843 | 10,265,586 |
|  | 1-oleoylglycerophosphoethanolamine | [HMDB11506](http://www.hmdb.ca/metabolites/HMDB11506) | 1,760,717 | 717,505 |
|  | 1-oleoylglycerophosphoinositol |  | 427,362 | 736,794 |
|  | 1-palmitoylglycerophosphocholine (16:0) | [HMDB10382](http://www.hmdb.ca/metabolites/HMDB10382) | 6,579,422 | 6,239,088 |
|  | 1-palmitoylglycerophosphoethanolamine | [HMDB11503](http://www.hmdb.ca/metabolites/HMDB11503) | 6,287,152 | 481,117 |
|  | 1-palmitoylglycerophosphoglycerol |  | 6,437,455 | 1,559,198 |
|  | 1-palmitoylglycerophosphoinositol | [HMDB61695](http://www.hmdb.ca/metabolites/HMDB61695) | 6,104,352 | 1,154,040 |
|  | 1-stearoylglycerophosphocholine (18:0) | [HMDB10384](http://www.hmdb.ca/metabolites/HMDB10384) | 487,947 | 734,759 |
|  | 1-stearoylglycerophosphoinositol | [HMDB61696](http://www.hmdb.ca/metabolites/HMDB61696) | 166,519 | - |
|  | 2-palmitoylglycerophosphocholine | [HMDB61702](http://www.hmdb.ca/metabolites/HMDB61702) | 367,344 | 334,454 |
|  | palmitoyl-oleoyl-glycerophosphoglycerol (2) |  | 519,265 | 266,693 |
| Medium Chain Fatty Acid | 5-dodecenoate (12:1n7) | [HMDB00529](http://www.hmdb.ca/metabolites/HMDB00529) | 610,748 | 2,283,128 |
|  | caprate (10:0) | [HMDB00511](http://www.hmdb.ca/metabolites/HMDB00511) | 4,638,353 | 9,519,592 |
|  | caproate (6:0) | [HMDB00535](http://www.hmdb.ca/metabolites/HMDB00535) | 1,101,043 | 1,972,581 |
|  | caprylate (8:0) | [HMDB00482](http://www.hmdb.ca/metabolites/HMDB00482) | 1,811,655 | 3,612,798 |
|  | heptanoate (7:0) | [HMDB00666](http://www.hmdb.ca/metabolites/HMDB00666) | 1,620,164 | 587,364 |
|  | laurate (12:0) | [HMDB00638](http://www.hmdb.ca/metabolites/HMDB00638) | 21,266,886 | 64,618,424 |
| Mevalonate Metabolism | 3-hydroxy-3-methylglutarate | [HMDB00355](http://www.hmdb.ca/metabolites/HMDB00355) | 31,241,704 | 16,946,286 |
| Monoacylglycerol | 1-linolenoylglycerol | [HMDB11569](http://www.hmdb.ca/metabolites/HMDB11569) | 3,450,437 | 9,402,899 |
|  | 1-linoleoylglycerol (1-monolinolein) |  | 21,300,358 | 230,424,096 |
|  | 1-myristoylglycerol (1-monomyristin) | [HMDB11561](http://www.hmdb.ca/metabolites/HMDB11561) | 460,374 | 2,894,156 |
|  | 1-oleoylglycerol (1-monoolein) | [HMDB11567](http://www.hmdb.ca/metabolites/HMDB11567) | 3,200,321 | 156,829,376 |
|  | 1-palmitoylglycerol (1-monopalmitin) | [HMDB31074](http://www.hmdb.ca/metabolites/HMDB31074) | 4,925,272 | 59,331,344 |
|  | 1-pentadecanoylglycerol (1-monopentadecanoin) |  | 266,202 | 587,997 |
|  | 2-linoleoylglycerol (2-monolinolein) | [HMDB11538](http://www.hmdb.ca/metabolites/HMDB11538) | 627,637 | 14,604,158 |
|  | 2-oleoylglycerol (2-monoolein) |  | 1,599,837 | 12,237,595 |
|  | 2-palmitoylglycerol (2-monopalmitin) | [HMDB11533](http://www.hmdb.ca/metabolites/HMDB11533) | 1,064,524 | 6,357,550 |
| Phosphatidylcholine | oleoyl-linoleoyl-glycerophosphocholine (1) |  | 4,122,321 | 3,183,703 |
|  | oleoyl-linoleoyl-glycerophosphocholine (2) |  | 1,462,078 | 704,027 |
|  | palmitoyl-linoleoyl-glycerophosphocholine (1) |  | 2,743,083 | 379,486 |
|  | palmitoyl-linoleoyl-glycerophosphocholine (2) |  | 6,176,508 | 1,744,728 |
|  | palmitoyl-oleoyl-glycerophosphocholine (1) |  | 1,427,999 | 672,909 |
| Phosphatidylinositol | oleoyl-linoleoyl-glycerophosphoinositol (1) |  | 901,792 | 1,397,967 |
|  | palmitoyl-linoleoyl-glycerophosphoinositol (1) |  | 5,876,814 | 2,985,819 |
| Phospholipid Metabolism | choline | [HMDB00097](http://www.hmdb.ca/metabolites/HMDB00097) | 599,931,008 | 722,152,256 |
|  | choline phosphate | [HMDB01565](http://www.hmdb.ca/metabolites/HMDB01565) | 14,146,123 | 31,528,856 |
|  | glycerophosphoethanolamine | [HMDB00114](http://www.hmdb.ca/metabolites/HMDB00114) | 375,492 | 1,438,771 |
|  | glycerophosphoinositol |  | 4,778,728 | 5,136,834 |
|  | glycerophosphorylcholine | [HMDB00086](http://www.hmdb.ca/metabolites/HMDB00086) | 30,075,690 | 235,681,840 |
| Polyunsaturated Fatty Acid (n3 and n6) | adrenate (22:4n6) | [HMDB02226](http://www.hmdb.ca/metabolites/HMDB02226) | 4,237,159 | 13,052,575 |
|  | dihomo-linoleate (20:2n6) | [HMDB05060](http://www.hmdb.ca/metabolites/HMDB05060) | 168,998 | 6,043,261 |
|  | dihomo-linolenate (20:3n3 or n6) | [HMDB02925](http://www.hmdb.ca/metabolites/HMDB02925) | 148,339 | - |
|  | docosadienoate (22:2n6) | [HMDB61714](http://www.hmdb.ca/metabolites/HMDB61714) | - | 470,588 |
|  | eicosapentaenoate (20:5n3) | [HMDB01999](http://www.hmdb.ca/metabolites/HMDB01999) | 340,610 | - |
|  | linoleate (18:2n6) | [HMDB00673](http://www.hmdb.ca/metabolites/HMDB00673) | 137,026,912 | 9,465,325,568 |
|  | linolenate [alpha or gamma; (18:3n3 or 6)] | [HMDB03073](http://www.hmdb.ca/metabolites/HMDB03073) | 92,844,024 | 1,212,979,072 |
| Secondary Bile Acid Metabolism | deoxycholate | [HMDB00626](http://www.hmdb.ca/metabolites/HMDB00626) | 245,714 | - |
| Short Chain Fatty Acid | valerate | [HMDB00892](http://www.hmdb.ca/metabolites/HMDB00892) | 512,490 | 731,165 |
| Sphingolipid Metabolism | sphinganine | [HMDB00269](http://www.hmdb.ca/metabolites/HMDB00269) | 86,719 | 1,265,766 |
| Steroid | 5alpha-pregnan-3beta,20beta-diol monosulfate (1) |  | 579,307 | 383,456 |
| Sterol | 4-cholesten-3-one | [HMDB00921](http://www.hmdb.ca/metabolites/HMDB00921) | - | 76,873 |
|  | campesterol | [HMDB02869](http://www.hmdb.ca/metabolites/HMDB02869) | 83,182 | 557,692 |
| **Nucleotide** | | | | |
| Purine and Pyrimidine Metabolism | methylphosphate | [HMDB61711](http://www.hmdb.ca/metabolites/HMDB61711) | - | 4,804,545 |
|  | allantoin | [HMDB00462](http://www.hmdb.ca/metabolites/HMDB00462) | 5,677,627 | 45,643,132 |
|  | hypoxanthine | [HMDB00157](http://www.hmdb.ca/metabolites/HMDB00157) | - | 296,190 |
|  | inosine | [HMDB00195](http://www.hmdb.ca/metabolites/HMDB00195) | 725,618 | 4,457,137 |
|  | xanthine | [HMDB00292](http://www.hmdb.ca/metabolites/HMDB00292) | 3,433,657 | 2,351,023 |
|  | xanthosine | [HMDB00299](http://www.hmdb.ca/metabolites/HMDB00299) | 2,829,760 | 684,372 |
| Purine Metabolism, Adenine containing | 1-methyladenine | [HMDB11599](http://www.hmdb.ca/metabolites/HMDB11599) | 3,572,021 | 4,436,731 |
|  | adenine | [HMDB00034](http://www.hmdb.ca/metabolites/HMDB00034) | 52,913,220 | 33,490,908 |
|  | adenosine | [HMDB00050](http://www.hmdb.ca/metabolites/HMDB00050) | 248,412,320 | 252,077,504 |
|  | adenosine 3',5'-cyclic monophosphate | [HMDB00058](http://www.hmdb.ca/metabolites/HMDB00058) | 116,257 | - |
|  | adenosine 5'-monophosphate | [HMDB00045](http://www.hmdb.ca/metabolites/HMDB00045) | 78,325,392 | 5,988,936 |
|  | N6-succinyladenosine | [HMDB00912](http://www.hmdb.ca/metabolites/HMDB00912) | 133,123 | 2,101,457 |
| Purine Metabolism, Guanine containing | 7-methylguanine | [HMDB00897](http://www.hmdb.ca/metabolites/HMDB00897) | 799,079 | - |
|  | guanosine | [HMDB00133](http://www.hmdb.ca/metabolites/HMDB00133) | 26,257,364 | 13,375,566 |
|  | N1-methylguanosine | [HMDB01563](http://www.hmdb.ca/metabolites/HMDB01563) | 145,521 | 226,924 |
|  | N2,N2-dimethylguanosine | [HMDB04824](http://www.hmdb.ca/metabolites/HMDB04824) | - | 86,624 |
| Pyrimidine Metabolism, Cytidine containing | cytidine | [HMDB00089](http://www.hmdb.ca/metabolites/HMDB00089) | 8,651,506 | 8,561,558 |
|  | cytosine | [HMDB00630](http://www.hmdb.ca/metabolites/HMDB00630) | 311,982 | 1,079,091 |
| Pyrimidine Metabolism, Orotate containing | dihydroorotate | [HMDB03349](http://www.hmdb.ca/metabolites/HMDB03349) | 54,087 | 83,752 |
|  | orotate | [HMDB00226](http://www.hmdb.ca/metabolites/HMDB00226) | 220,015 | 579,924 |
| Pyrimidine Metabolism, Uracil containing | 2'-deoxyuridine | [HMDB00012](http://www.hmdb.ca/metabolites/HMDB00012) | 89,195 | - |
|  | 4-ureidobutyrate |  | 23,173,338 | 1,333,347 |
|  | 5-methyluridine (ribothymidine) | [HMDB00884](http://www.hmdb.ca/metabolites/HMDB00884) | - | 75,035 |
|  | beta-alanine | [HMDB00056](http://www.hmdb.ca/metabolites/HMDB00056) | 374,733 | 246,109 |
|  | N-acetyl-beta-alanine |  | 721,809 | 894,407 |
|  | pseudouridine | [HMDB00767](http://www.hmdb.ca/metabolites/HMDB00767) | 137,876 | 3,217,247 |
|  | uracil | [HMDB00300](http://www.hmdb.ca/metabolites/HMDB00300) | 120,433 | 351,026 |
|  | uridine | [HMDB00296](http://www.hmdb.ca/metabolites/HMDB00296) | 15,209,662 | 20,129,712 |
|  | isoleucylglycine |  | - | 453,871 |
|  | valylglutamine |  | - | 554,544 |
|  | valylleucine |  | 81,889 | 4,001,783 |
| Gamma-glutamyl Amino Acid | gamma-glutamyl-2-aminobutyrate |  | 4,909,089 | 1,425,246 |
|  | gamma-glutamylalanine | [HMDB29142](http://www.hmdb.ca/metabolites/HMDB29142) | 1,173,894 | 9,018,161 |
|  | gamma-glutamylglutamate | [HMDB11737](http://www.hmdb.ca/metabolites/HMDB11737) | 1,865,509 | 1,271,113 |
|  | gamma-glutamylglutamine | [HMDB11738](http://www.hmdb.ca/metabolites/HMDB11738) | 312,071 | 3,280,159 |
|  | gamma-glutamylisoleucine | [HMDB11170](http://www.hmdb.ca/metabolites/HMDB11170) | 5,561,916 | 805,042 |
|  | gamma-glutamylleucine | [HMDB11171](http://www.hmdb.ca/metabolites/HMDB11171) | 419,273,760 | 512,326 |
|  | gamma-glutamylmethionine | [HMDB29155](http://www.hmdb.ca/metabolites/HMDB29155) | 10,306,020 | 603,857 |
|  | gamma-glutamylphenylalanine | [HMDB00594](http://www.hmdb.ca/metabolites/HMDB00594) | 4,832,061 | 879,103 |
|  | gamma-glutamylthreonine | [HMDB29159](http://www.hmdb.ca/metabolites/HMDB29159) | 197,085 | 176,494 |
|  | gamma-glutamyltryptophan | [HMDB29160](http://www.hmdb.ca/metabolites/HMDB29160) | 2,153,754 | 1,366,673 |
|  | gamma-glutamyltyrosine | [HMDB11741](http://www.hmdb.ca/metabolites/HMDB11741) | 458,681 | 566,542 |
|  | gamma-glutamylvaline | [HMDB11172](http://www.hmdb.ca/metabolites/HMDB11172) | 5,477,714 | 5,105,156 |
| **Phytochemicals/Other** | | | | |
| Benzoate Metabolism | 2-hydroxyhippurate (salicylurate) | [HMDB00840](http://www.hmdb.ca/metabolites/HMDB00840) | - | 86,256 |
|  | benzoate | [HMDB01870](http://www.hmdb.ca/metabolites/HMDB01870) | 4,307,687 | 5,354,217 |
| Chemical | N-methylpipecolate |  | 64,955,940 | 7,209,496 |
|  | O-sulfo-L-tyrosine |  | 78,572 | 484,287 |
|  | sulfate | [HMDB01448](http://www.hmdb.ca/metabolites/HMDB01448) | 27,710,566 | 10,203,519 |
| Drug/Plant | 6-oxopiperidine-2-carboxylic acid |  | 3,627,378 | 2,112,532 |
|  | salicylate | [HMDB01895](http://www.hmdb.ca/metabolites/HMDB01895) | 308,467 | 8,155,134 |
| Food Component/Plant | 2,3-dihydroxyisovalerate | [HMDB12141](http://www.hmdb.ca/metabolites/HMDB12141) | 217,133 | 491,428 |
|  | 2-isopropylmalate | [HMDB00402](http://www.hmdb.ca/metabolites/HMDB00402) | 1,599,339 | 125,371 |
|  | 2-piperidinone |  | 51,866,064 | 43,266,232 |
|  | ergothioneine | [HMDB03045](http://www.hmdb.ca/metabolites/HMDB03045) | 2,149,285 | - |
|  | erythritol | [HMDB02994](http://www.hmdb.ca/metabolites/HMDB02994) | - | 285,837 |
|  | gluconate | [HMDB00625](http://www.hmdb.ca/metabolites/HMDB00625) | 6,867,422 | 32,796,584 |
|  | homostachydrine | [HMDB33433](http://www.hmdb.ca/metabolites/HMDB33433) | 192,234 | 784,996 |
|  | indolin-2-one |  | 427,580 | 1,449,349 |
|  | quinate | [HMDB03072](http://www.hmdb.ca/metabolites/HMDB03072) | 246,228 | 10,826,823 |
|  | stachydrine | [HMDB04827](http://www.hmdb.ca/metabolites/HMDB04827) | 3,451,568 | 35,157,772 |

Alpha-CEHC, 2, 5, 7, 8-tetramethyl-2-(2'-carboxyethyl)-6-hydroxychroman; ADMA, asymmetric dimethylarginine; Ala, alanine; Arg, arginine; Asp, aspartate; BCAA = branched-chain amino acid; Cys, cysteine; 9,10-diHOME, (12Z)-9,10-dihydroxyoctadec-12-enoic acid; 12,13-diHOME; 12,13-dihydroxy-9Z-octadecenoic acid; Glu, glutamate; Gly, glycine; His, histidine; 9-HODE, 13-hydroxyoctadecadienoic acid; 13-HODE, 13-hydroxyoctadecadienoic acid; HMDB, Human Metabolome Database; Ile, isoleucine; Leu, leucine; Lys, lysine; Met, methionine; Phe, phenylalanine; Pro, proline; SAM, S-adenosyl methionine; SDMA, symmetric dimethylarginine; Ser, serine; TCA, tricarboxylic acid; Thr, threonine; Trp, tryptophan; Tyr, tyrosine; Val, valine.
